# Structure-centered design of lipid-based nanocarriers to overcome biological barriers

**DOI:** 10.1016/j.mtbio.2025.102425

**Published:** 2025-10-20

**Authors:** Yeeun Woo, Jinwook Yoon, Yoseph Seo, Yunseon Han, Hah Young Yoo, Hiesang Sohn, Min-Ho Lee, Taek Lee

**Affiliations:** a Department of Chemical Engineering, Kwangwoon University, 20 Gwangwoon-Ro, Nowon-Gu, Seoul, 01897, Republic of Korea; b Department of Biotechnology, Sangmyung University, 20, Hongjimun 2-gil, Seoul, 03016, Republic of Korea; c School of Integrative Engineering, Chung-Ang University, Heukseok-dong, Dongjak-gu, Seoul, 06910, Republic of Korea

**Keywords:** Drug delivery systems, Lipid-based nanoparticles, Biological barriers, Structure-centered design, Therapeutic outcomes

## Abstract

As technology has evolved in response to pressing human challenges, drug delivery systems (DDSs) have also evolved to overcome complex biological barriers. Particularly, the continuous development of therapeutic strategies has enabled the integration of nanotechnology, leading to the emergence of diverse nanoscale DDSs. Concerns about the potential toxicity of nanomedicines diverted attention to lipid-based nanocarriers (LBNs), regarded as safer and more biocompatible platforms. In this review, the structural evolution of LBNs is categorized into three stages according to the increasing functional complexity required to address biological barriers, providing a framework to understand how designs advanced with physiological demands. This trajectory encompasses emulsions and LBNs, with each stage advanced by strategies such as lipid composition tuning, surface functionalization, biomimetic construction, and hybridization with other platforms. Over a few decades, this transition has described that the progression of LBNs reflects not merely chronological advances but a stepwise evolution of structural adaptations shaped by biological barriers. This analytical perspective is presented through a comprehensive discussion of the structural strategies utilized in recent LBN research. Consequently, structural evolution provides a foundation for refining design approaches in drug delivery and achieving improved therapeutic outcomes.

## Highlights

• Drug delivery systems have evolved in response to challenges in biological barriers.
• The structure of nanocarriers is essential for the coordination of their functions.
• Structural design is an expression of design intent to overcome biological barriers.
• Structure-centered designs are key to the development of next-generation DDSs.

## Graphical abstract

**Figure:**
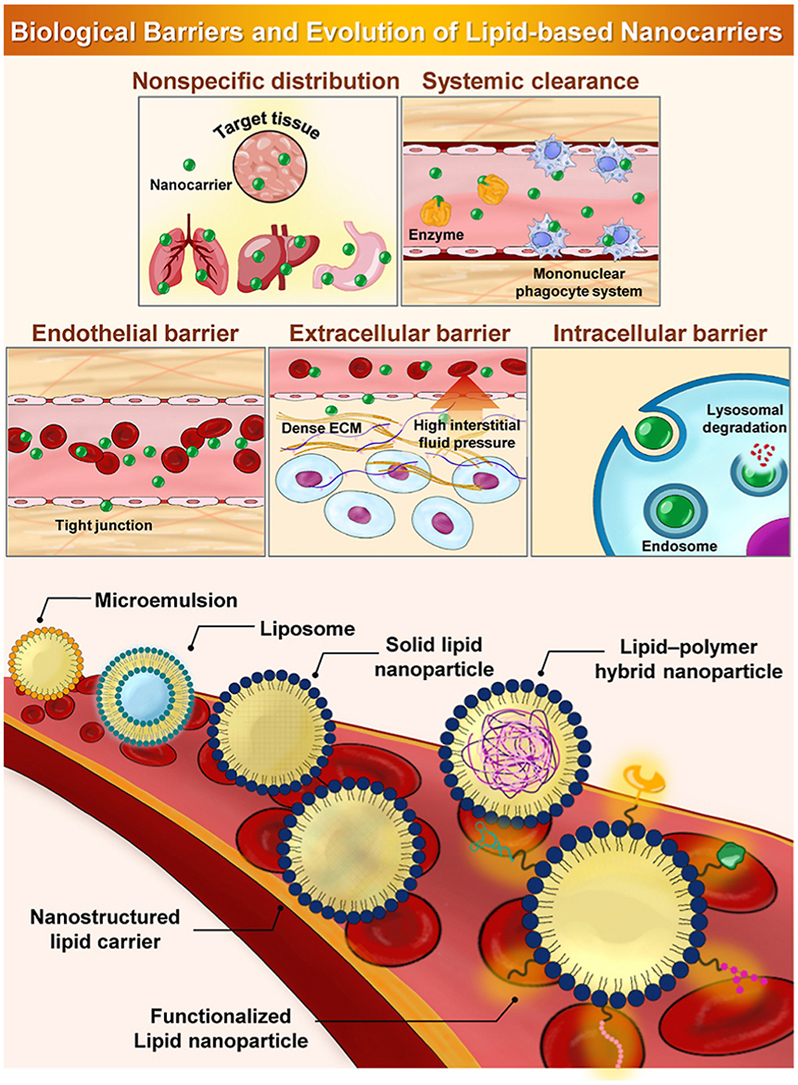

## 1 Introduction

Drug delivery systems (DDSs), developed to enhance therapeutic efficacy and ensure *in vivo* stability, have steadily evolved through advances in formulation design. This progression is driven by the persistent need to treat diseases and exert precise control over pathological conditions [1]. Structural modifications have served as a pivotal strategy for overcoming biological barriers, such as immunogenicity under *in vivo* conditions, by equipping carriers with specific functional attributes [2,3]. Currently, most DDS research focuses on the implementation of structural engineering strategies to improve the bioavailability of therapeutics [[4], [5], [6]]. Early drug formulation was limited by rapid clearance by the liver and kidneys and strong immune responses, which significantly reduced circulation time and therapeutic outcomes [7]. To address these challenges, functional polymers, such as polyethylene glycol (PEG), were covalently attached to drug molecules to prolong retention time and reduce immune recognition and renal elimination [8]. Non-specific distribution, which often led to cytotoxicity in off-target tissues, was partially alleviated through the use of PEG-based drug conjugates that incorporated targeting antibodies [9]. However, the increasing demand for greater precision, such as that related to membrane permeability, controlled release, and bio-adhesion, has revealed the limitations of simple conjugation strategies and has underscored the need for more advanced therapeutic systems [10,11].

**Fig. 1:**
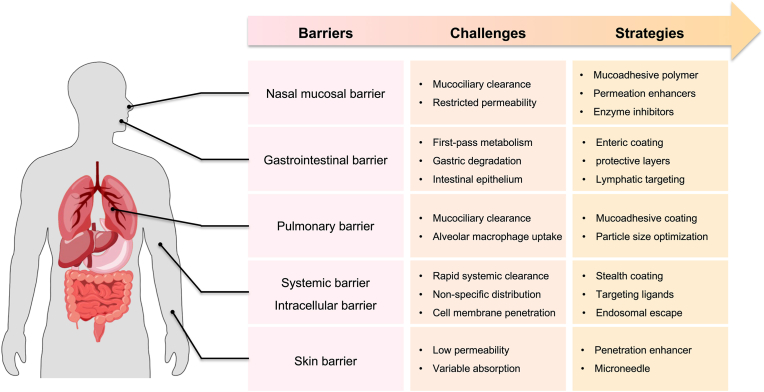
Representative examples of administration routes with their biological barriers, delivery challenges, and strategies in drug delivery system.

Each administration route is limited by distinct biological barriers. For oral delivery, enzymatic degradation in the gastrointestinal tract significantly impairs drug stability. For intravenous delivery, the vascular endothelium and the mononuclear phagocyte system (MPS) reduce systemic circulation. Tumor environments exhibit a dense extracellular matrix (ECM) and high interstitial pressure, both of which hinder drug penetration. For intracellular targeting, efficient endosomal escape remains a critical challenge [3,12,13]. Thus, DDS development has increasingly focused on nanoscale carriers that encapsulate drugs and provide physicochemical stability, enhanced membrane permeability, and protection from degradation [14]. For instance, orally delivered drugs often suffer from instability in acidic and enzymatic environments, poor mucosal permeability, and extensive first-pass metabolism, resulting in reduced bioavailability [15,16]. To overcome these barriers, negatively charged surface-modified nanocarriers have been designed to facilitate mucosal penetration and promote systemic distribution via the lymphatic system [17]. As understanding of physiological environments deepens and technical capabilities expand, new functional demands for DDS design continue to emerge. At the same time, these examples show how the physiological challenges associated with each administration route directly shape the structural design requirements of carriers [[18], [19], [20]] (Fig. 1).

DDSs have progressively evolved to respond to these challenges through structural innovation, and material selection has shifted toward biocompatible and safer alternatives. While early nanocarrier systems employed materials such as carbon, silica, and noble metals (e.g., gold and silver), growing concerns over cytotoxicity have led to the increased use of lipid- and chitosan-based materials [[21], [22], [23], [24]] (Table 1). Among these, lipid-based nanocarriers (LBNs), composed of lipids and surfactants, have garnered attention owing to their biological compatibility and structural flexibility [[25], [26], [27]]. Their development reflects a sequence of adaptive refinements. Microemulsions improved the solubility of hydrophobic compounds but provided little control under physiological conditions. Liposomes, based on phospholipid bilayers, allowed simultaneous loading of hydrophilic and hydrophobic drugs and offered good biocompatibility, yet instability and burst release remained problematic. Solid lipid nanoparticles (SLNs) introduced crystalline lipid matrices that improved stability and reduced premature leakage, but their rigidity limited flexibility in drug incorporation. To overcome this limitation, nanostructured lipid carriers (NLCs) combined solid and liquid lipids, forming an amorphous matrix with greater versatility in drug loading and more consistent release profiles. More recently, surface modifications with polyethylene glycol (PEG), zwitterionic coatings, or targeting ligands have been adopted to extend circulation, reduce immune clearance, and achieve site specific accumulation [[28], [29], [30]].

**Table 1:** Comparative features of representative nanocarrier platforms.

Criterion	Polymeric nanoparticles	Inorganic nanoparticles	Exosomes/Extracellular vesicles	LBNs
Loading & release control	Programmable release via degradable polymer matrices	Stimuli-responsive release (magnetic, light, ultrasound, pH)	Natural cargo exchange with intrinsic selectivity	Highly tunable release through lipid composition and architecture
Biocompatibility/Immunogenicity	Compatibility depends on polymer chemistry	Useful for imaging; responsive to external triggers	Low innate immunogenicity; immune modulation potential	Clinically validated lipid components with proven safety and biocompatibility
Scalability & Manufacturability	Adaptable to GMP-oriented synthesis	Established large-scale synthesis methods	Biological origin provides functionality	Standardized, modular, and scalable manufacturing processes supported by regulatory experience
Clinical/Commercial proximity	Several polymer–drug conjugates and depots approved	Widely used in diagnostics	Early clinical studies underway	Multiple therapeutic approvals; ionizable LNPs validated in mRNA vaccines
Functional versatility	Broad design space; adjustable degradation and release	Strong theranostic platforms	Intrinsic tropism; natural intercellular communication	Versatile delivery of nucleic acids, small molecules, peptides, and vaccines with clinical success
Limitation	Batch-to-batch variability; possible residual toxicity	Non-biodegradability causing long-term accumulation; limited therapeutic translation	Source heterogeneity; poor scalability and lack of standardization	Potential immune responses under certain conditions stability challenges for sensitive payload
Reference	[211,212]	[213,214]	[215,216]	[217,218]

Conventional DDS classifications typically rely on material composition, morphology, or manufacturing technique, often overlooking the functional rationale embedded in structural choices. While earlier reviews focused on technical taxonomy, this review reframes the structural evolution of DDS as a strategic response to physiological barriers, emphasizing structure as a vessel of function. Special attention has been given to LBNs as representative biocompatible platforms, examining how specific structural modifications have been employed to overcome delivery constraints. Finally, the discussion considers how emerging demands in next-generation DDSs call for function-oriented interpretation and strategically guided structural design. To ensure comprehensive coverage, the literature was collected from SCIE-indexed journals (2015–2025) through PubMed, Web of Science, and Scopus, with most references concentrated after 2020 when structural innovations became more active and covering topics such as lipid-based nanocarriers, physiological barriers in drug delivery, and next-generation DDSs.

## 2 Stage-wise structural evolution of lipid-based nanocarriers

The structural forms of DDSs have diversified based on the biological constraints encountered during drug delivery and the resulting functional demands [31,32]. For example, each administration route presents distinct loss mechanisms and barrier types, requiring delivery systems to adopt different architectural strategies for effective transport (Table 2). After administered, drug molecules or carriers face pharmacokinetic limitations. Low solubility and aggregation from ionic interactions and protein binding impair dispersion and destabilize colloids. Once in systemic circulation, plasma proteins rapidly adsorb onto the carrier surface, inducing opsonization. This process labels the carrier as foreign and promotes its recognition and elimination by immune cells. Drugs may also be excluded by renal filtration or hepatic metabolism, leading to rapid clearance and a sharp decline in systemic exposure. These consecutive challenges serve as fundamental constraints in DDS design [33].

**Table 2:** Physiological barriers and drug delivery challenges by administration route.

Administration Route	Physiological barrier	Barriers to drug delivery	Delivery strategy	Limitation	Reference
Ocular	Tear turnover and corneal penetration barrier	Tear turnover, corneal barrier, enzymatic degradation, rapid drainage	Mucoadhesive and viscous formulation strategies; prolongation of ocular retention	Poor penetration into posterior segment; frequent dosing needed	[219,220]
Intranasal	Nasal mucosal and enzymatic barrier	Mucosal clearance, enzymatic degradation, tight junctions, short residence time	Mucoadhesive polymers and surface modification for prolonged retention	Too short residence time with small dosing volume; limited brain penetration	[[221], [222], [223]]
Oral	Gastrointestinal stability and first-pass metabolism	Enzymatic degradation, low gastric pH, mucosal clearance, efflux transporters, poor absorption	Drug protection using nanocarriers; mucoadhesive coatings; pH-responsive formulations for intestinal absorption	Variable absorption across patients; instability in GI fluids; limited bioavailability for macromolecules	[224,225]
Inhalation	Mucociliary and alveolar clearance barrier	Mucociliary clearance, enzymatic degradation, deposition inefficiency, macrophage uptake	Enhancement of pulmonary deposition and clearance avoidance; lesion-specific delivery via targeting or stimuli-responsive release	Rapid clearance with heterogeneous deposition; potential immune activation	[226,227]
Intravenous	Systemic clearance and tissue penetration barrier	MPS clearance, non-specific distribution, renal clearance, tumor penetration, cellular uptake, endosomal escape	Stealth design; active targeting ligands; immune evasion through biomimetic coatings	Risk of rapid clearance; off-target toxicity; limited penetration into dense tumor tissues	[228,229]
Subcutaneous/Intramuscular	Interstitial transport and lymphatic barrier	Enzymatic degradation at injection site, poor lymphatic absorption, slow diffusion	Enhancement of enzymatic stability and lymphatic uptake via nanocarriers with interstitial diffusivity	Lymphatic uptake inefficient; inefficient and variable systemic exposure	[230,231]
Transdermal	Stratum corneum and skin permeability barrier	Low permeability, enzymatic activity, variability in skin condition	Enhancement of skin permeation and drug protection	Hydrophilic or large molecules show low absorption with strong inter-patient variability	[232,233]
Rectal/Vaginal	Mucosal and pH variability barrier	Mucosal clearance, enzymatic degradation, pH variability, limited absorption area	Application of pH-responsive nanocarriers for improved retention and controlled release	Local irritation; variable absorption with pH and mucus changes	[234]

**Fig. 2:**
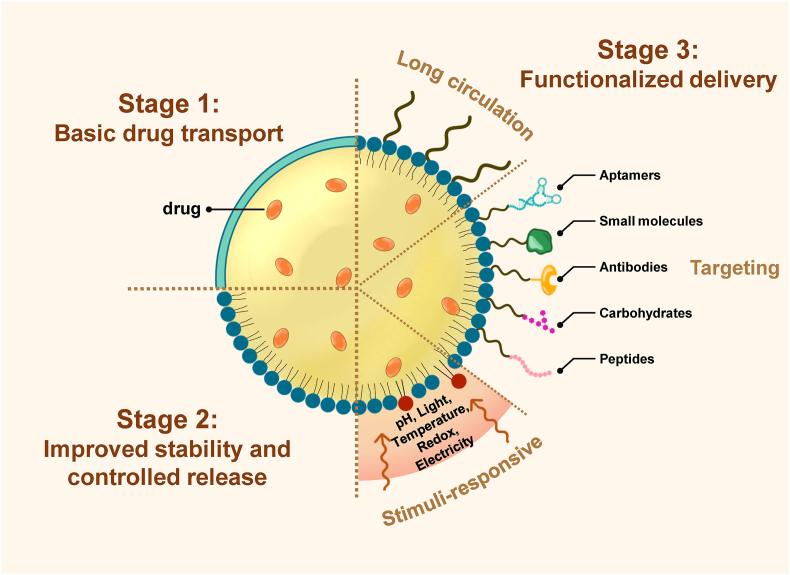
Progressive advancement of structural functionality in lipid-based nanocarriers.

To systematically understand how DDSs have structurally adapted to these challenges, this section categorizes their evolution into three stages based on the increasing level of functional complexity required to overcome physiological barriers. An overview of these three stages is provided in Table 3. Initially, DDSs focused on simple encapsulation and transport. Later designs emphasized prolonged exposure through enhanced stability and controlled release. At a more advanced stage, carriers have incorporated immune evasion, targeting specificity, and responsiveness to physiological stimuli [34,35]. This staged classification illustrates how delivery architectures have been iteratively optimized in response to evolving functional demands becomes possible (Fig. 2).

**Table 3:** Stage-wise guide to lipid-based nanocarriers by functional complexity.

Stage	Target function	Key design strategy	Remaining limitations	Examples
Basic drug transport	Drug encapsulation and solubility enhancement	Surfactant-stabilized dispersions, Lipid bilayer vesicles	Structural instability, rapid clearance, premature drug leakage, particle aggregation	Microemulsions, nanoemulsions, early liposomes
Stability and controlled release	Improved stability and reduced initial burst release	Solid lipid matrices, hybrid interfaces	Incomplete release control, Nonspecific distribution	SLN, NLC, lipid-polymer hybrids
Functionalized delivery	Extend circulation, targeting, stimuli- responsiveness	Stealth coatings (PEGylation, zwitterionic), targeting ligands, tumor microenvironment–responsive systems	Immune adaptation, Tumor heterogeneity, unpredictable drug release, formulation complexity	PEGylated LNPs, ligand-modified liposomes, pH/ROS/enzyme-sensitive liposomes

### 2.1 Stage 1: structures focused on basic transport function

The earliest DDS forms were primarily designed to facilitate delivery of bioactive agents into the body [36,37]. To address pharmacokinetic limitations under biological conditions, these systems focused on stabilizing drug molecules and promoting their effective distribution throughout the system [38]. Despite their structural simplicity, these carriers often achieved satisfactory outcomes by compensating for the inherent physicochemical limitations of drugs. During this initial stage, design strategies were mostly oriented toward improving the systemic availability of poorly soluble agents [39]. A representative approach involved the use of surfactants to form stable emulsions, enabling the dispersion of hydrophobic drugs within immiscible phases, thereby enhancing their solubility [40,41]. Another widely implemented approach involved the use of phospholipid bilayers to construct liposomes capable of encapsulating both hydrophilic and hydrophobic substances within a unified structure [42,43].

#### 2.1.1 Microemulsion/nanoemulsion

**Fig. 3:**
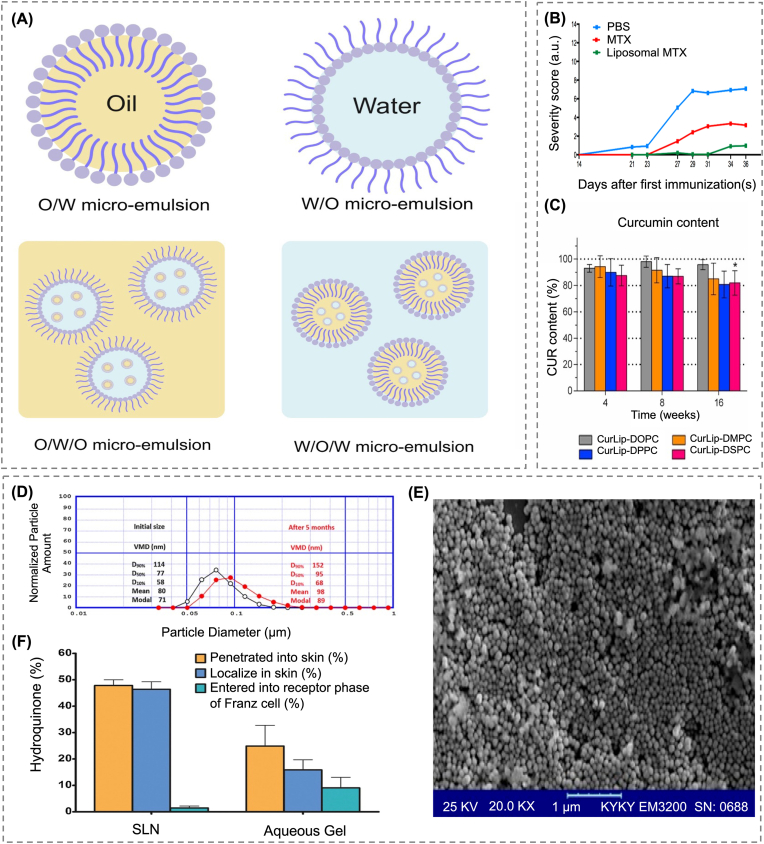
(A) Schematic illustration of O/W, W/O, O/W/O, and W/O/W microemulsion formulation. Created with BioRender.com. (B) Comparative analysis of arthritis severity among liposomal MTX, Non-liposomed MTX, and PBS treatment groups [64]. Copyright 2022, Multidisciplinary Digital Publishing Institute. (C) Stability of curcumin content in various liposomal formulations during storage at 4–8 °C for up to 16 weeks (mean ± SD, n = 3–4) [66]. Copyright 2019, Elsevier. (D) Particle size distribution of HQ-SLNs, demonstrating consistent VMD immediately after preparation and after 5 months of storage. (E) SEM image showing spherical morphology and uniform structure of HQ-SLNs. (F) Distribution of hydroquinone in rat skin, including deposition and penetration into the stratum corneum, following treatment with HQ-SLN and conventional hydrogel treatment [85]. Copyright 2015, Elsevier.

The increasing prevalence of poorly water-soluble drugs in pharmaceutical development has highlighted solubility enhancement as a fundamental challenge in DDS design [44]. One of the earliest strategies implemented to address this involved physical dispersion of drugs to facilitate their absorption [45]. Among these approaches, emulsion-based systems garnered particular attention owing to their structural simplicity and efficacy in improving the solubility of hydrophobic compounds [46,47]. These systems can be formulated in various configurations, including oil-in-water (O/W), water-in-oil (W/O), water-in-oil-in-water (W/O/W), and oil-in-water-in-oil (O/W/O) formulations, and are advantageous for practical application owing to their relatively simple preparation processes (Fig. 3A) [48,49]. The core principle involves dissolving a hydrophobic drug in an oil phase and dispersing them into the aqueous medium through surfactant-mediated emulsification. In this process, surfactants are characterized by their hydrophilic-lipophilic balance (HLB) value, where low HLB surfactants stabilize the oil droplets and high HLB surfactants stabilize the aqueous phase, thereby enabling uniform drug incorporation within the interfacial structures [50].

For instance, Aloisio et al. developed a biocompatible O/W-type microemulsion using soybean oil, soy phosphatidylcholine, sodium oleate, and Eumulgin® HRE40 for the oral delivery of the poorly soluble drugs, sulfamerazine (SMR) and indomethacin (INM) [51], achieving solubility levels of 22.0 and 62.3 mg/mL, respectively, for the drugs. The emulsion exhibited favorable physical stability, with particle sizes ranging from 100 to 177 nm and zeta potentials ranging from −52 to −66 mV. Polarized light microscopy revealed a uniform liquid phase, and NMR analyses along with partition coefficient measurements confirmed drug distribution within the oil domain. Of note, SMR exhibited significantly enhanced solubility and release rates in the microemulsion environment. This system exemplifies a formulation focused on solubility enhancement through physical stabilization rather than advanced structural features.

In cases where water-soluble drugs exhibit limited absorption due to instability or low permeability in the gastrointestinal tract, similar emulsions can also be effective. Xu et al. developed a W/O-type microemulsion for the oral delivery of troxerutin (TX), a water-soluble flavonoid with poor intestinal absorption [52]. The system, composed of lecithin, ethanol, isopropyl myristate, and water, formed particles of approximately 50 nm in size. *In vitro* studies showed a 2.8-fold increase in cellular permeability as compared to that of an aqueous solution, and *in vivo* testing demonstrated an over 200 % improvement in bioavailability, indicating effective barrier penetration via physical encapsulation alone.

A similar strategy was employed by Jang et al., who formulated a W/O/W-type nanoemulsion encapsulating methotrexate (MTX), a widely used anticancer and immunomodulatory agent limited by poor bioavailability, a short half-life, and tissue toxicity [53]. Olive oil was used as the oil phase in the formulation, while labrasol and ethanol were used as the surfactant and co-surfactant, respectively. This formulation exhibited a mean particle size of 173.77 nm, a zeta potential of −35.63 mV, and an encapsulation efficiency of 90.37 %. *In vitro* experiments showed accelerated dissolution and release rates for the nanoemulsion as compared to free MTX; in addition, *in vivo* studies revealed a three-fold increase in bioavailability and improved lymphatic drug accumulation, demonstrating the potential of structural modulation for redirecting absorption pathways and targeting lymphatic tissues.

#### 2.1.2 Liposomes

Early drug administration routes, including oral and transdermal formulations, often failed to achieve adequate therapeutic outcomes due to poor absorption, rapid clearance, and extensive first-pass metabolism [54]. Although convenient, these forms frequently resulted in insufficient drug accumulation at target tissues and required higher doses, thereby increasing systemic side effects [55]. To overcome such limitations, parenteral delivery became essential, as it bypasses absorption barriers and provides more reliable systemic exposure [56]. However, even injectable formulations exhibited notable drawbacks. Emulsion-based systems, one of the earliest parenteral drug delivery approaches, displayed physicochemical instability. Lipophilic components tended to diffuse from smaller to larger droplets (Ostwald ripening), progressively disrupting size uniformity and causing phase separation. In addition, residual organic solvents and high surfactant concentrations introduced during formulation raised toxicity concerns, including vascular irritation and hepatic injury [57,58]. These limitations underscored the demand for delivery vehicles capable of maintaining stability in biological environments while reducing formulation-related toxicity.

To address these challenges, liposomes, vesicular carriers composed of phospholipid bilayers, were introduced as structural solutions [59]. Their biocompatibility, derived from their similarity to biological membranes, and their high drug-loading capacity made them highly promising delivery platforms [43]. Their hydrophilic core encapsulates water-soluble agents, while their lipid bilayer incorporates lipophilic compounds, enabling broad application across diverse drug types [60]. Despite their relatively simple structure, liposomes protect drugs during circulation and modulate their release, thereby alleviating the instability and toxicity concerns that associated with earlier systems [61]. Of note, their ability to remain stably dispersed in biological environments without the need for additional surface modifications positioned them as a foundational platform for early DDS development [62].

For instance, small, hydrophilic molecules can be loaded into the aqueous core of liposomes, with delivery performance improved through the adjustment of lipid composition and the optimization of manufacturing conditions [63]. Guimarães et al. encapsulated MTX, a drug widely used for the management of autoimmune disorders, in liposomes to circumvent its poor bioavailability and resistance susceptibility [64]. They employed an ethanol injection method combined with a pre-concentration strategy that involved reducing the aqueous phase volume and adjusting the solvent ratio. This approach yielded an encapsulation efficiency of approximately 12 % without any surface modification. In mouse models of arthritis, liposomal MTX demonstrated superior therapeutic effects to free MTX (Fig. 3B), indicating that improvements in composition and processing alone can significantly enhance drug delivery performance.

Similarly, Soriano-Romaní et al. developed an ophthalmic liposomal formulation containing the hydrophilic peptide, KRFK (Lys-Arg-Phe-Lys), derived from thrombospondin-1 [65]. The liposomes were composed of phosphatidylcholine, cholesterol, and vitamin E, and were prepared using a solvent evaporation technique. They exhibited physicochemical properties suitable for eye drop formulation, including the appropriate pH, osmolality, and particle size, inducing no cytotoxicity in human corneal epithelial cells. In porcine corneal models, KRFK-loaded liposomes produced stronger fluorescence signals than the free peptide, and were detected in deeper corneal layers after 60 min of exposure. These results suggest that liposomes can improve the tissue permeability and local retention of water-soluble biomolecules.

Liposomes have also been used to deliver lipophilic drugs by directly incorporating them into their lipid membrane. Kolter et al. assessed the delivery efficiency and stability of curcumin, a poorly soluble compound, using liposomes with various phospholipid compositions [66]. Among these, the formulation with dioleoylphosphatidylcholine (DOPC), known for its high membrane fluidity, achieved approximately 95 % encapsulation efficiency and exhibited excellent stability during storage, effectively preventing curcumin degradation (Fig. 3C). The optimized formulation consisted of DOPC (65 mol%), cholesterol (30 mol%), and DSPE-mPEG2000 (5 mol%), yielding nanoparticles with an average size of 130 ± 12 nm, a low PDI of 0.07 ± 0.02, and an incorporation efficiency of 95 ± 4 %. These liposomes also retained high drug levels under dilution and physiological conditions, supporting their applicability in the formulation of unstable hydrophobic drugs. Biological performance was assessed using three-dimensional tumor spheroids derived from LS neuroblastoma cells. While liposomal curcumin exhibited cytotoxicity levels comparable to those of the free drug in two-dimensional cultures, its effectiveness decreased in the 3D model, possibly due to drug leakage or modified interactions within the complex environment. These observations emphasize the need for further structural refinement to improve drug retention and tissue penetration.

Beyond systemic and anticancer applications, liposomes have also been explored for ocular drug delivery. To overcome the rapid degradation and poor corneal permeability of unencapsulated ascorbic acid (AA), Csorba et al. synthesized a lyophilized liposomal formulation of AA (AA-LLipo) [67]. The liposomal formulation of AA-LLipo was prepared using soy phosphatidylcholine and was lyophilized to stabilize the formulation, and had desirable physicochemical characteristics, with a mean particle size of 80–100 nm with a uniform distribution and negative zeta potential. Compared to the AA-LLipo, the AA encapsulated vesicles extended the half-life of AA from 73 min to 210 min at 35 °C, indicative of an increase in chemical stability and degradation under physiological conditions were less. In a parallel artificial membrane permeability assay (PAMPA), it was demonstrated that the AA-LLipo was significantly more permeable compared to free AA, especially when simulating damaged/inflamed corneal epithelium. *Ex vivo* studies with porcine eyes confirmed an increase in drug accumulation in porcine cornea, and Raman mapping of the porcine cornea indicated an enhanced penetration into stroma compared to free AA. The release profile of AA-LLipo was similar AA frees, however, with the ocular surface retention time being increase efficacy was seen in improving ocular drug delivery.

### 2.2 Stage 2: structures reinforced for stability and controlled release

Emulsion-based systems improved drug solubility but still represented a thermodynamically unstable system as Ostwald ripening and droplet coalescence led to phase separation and burst release [68]. Their passive diffusion–driven mechanism also limited sustained or environment-responsive delivery, while the need for high surfactant and co-solvent concentrations often introduced formulation-related toxicity [69,70]. Liposomes offered biocompatibility and versatile drug loading, yet rapid clearance from protein corona formation and complement activation, together with insufficient endosomal escape frequently resulting in lysosomal degradation, restricted their therapeutic efficacy [71]. Earlier phospholipid-based liposomes provided effective short-term drug protection and transport, but their performance was limited under *in vivo* conditions [72]. A common obstacle is particle aggregation in physiological fluids, which can increase particle size and alter surface properties, often leading to rapid clearance or decreased tissue penetration [73]. Their liquid-core configuration is highly sensitive to dilution and interactions with serum proteins, which contribute to structural destabilization and premature drug leakage [74]. Insufficient retention of hydrophobic drugs further impedes their therapeutic efficacy [64]. To address these issues, structurally reinforced carriers have been developed to preserve integrity under biological conditions and minimize premature release [75]. Solid lipid nanoparticles (SLN), nanostructured lipid carriers (NLC), and lipid-polymer hybrid nanoparticles (LPN) represent key advances in this direction. These platforms mark a pivotal shift in DDS evolution, where the design focus expands beyond mere transport efficiency to incorporating physical robustness and precise control over drug kinetics.

#### 2.2.1 Solid lipid nanoparticle (SLN)

Irrespective of the drug type they carry or its physicochemical characteristics, nanocarriers are constantly faced with diverse biological challenges that compromise their structural stability [76]. This instability can lead to premature drug release or even carrier disintegration, underscoring *in vivo* stability as a crucial objective in nanocarrier design [77]. To address this persistent challenge, solid lipid nanoparticles (SLNs) were developed using lipids that remain solid at room temperature [[78], [79], [80]]. SLNs leverage the crystalline structures of solid lipids to suppress unintended drug leakage; in addition, their surfaces are stabilized by surfactants, ensuring physical integrity and a uniform particle size under physiological conditions [81]. Furthermore, via precise tuning of lipid composition and fabrication parameters, SLNs offer significant design flexibility in controlling drug loading capacity, release kinetics, and physicochemical properties [82]. These inherent features position SLNs as early but important examples of structural optimization aimed at overcoming the physical limitations of earlier-generation carriers.

For instance, Baek et al. developed a paclitaxel (PTX)-loaded SLN formulation incorporating 2-hydroxypropyl-β-cyclodextrin (HPCD) for enhanced physical stability [83]. This system, termed, PSC (PTX-loaded SLN with cyclodextrin), was composed of stearic acid as the solid lipid, with lecithin and Poloxamer 188 as stabilizers, and an encapsulation efficiency of 78.2 % compared with 71.1 % for the non-cyclodextrin formulation. PSC maintained a stable particle size and polydispersity index (PDI) during storage for 180 days at both 25 and 40 °C. Of note, PSC retained its initial structural properties even under heat stress, demonstrating robust stability at elevated temperatures. Release studies showed that PSC reduced the initial burst effect (32.6 % within 2 h) compared with conventional SLNs (43.2 %), while still supporting sustained cumulative release over 24 h. Under plasma-mimicking conditions, the formulation exhibited minimal changes in particle size and dispersion, and drug release remained steady. Cytotoxicity studies carried out using the multidrug-resistant MCF-7/ADR breast cancer cell line confirmed that under long-term storage conditions, PSC preserved the therapeutic effects of PTX, highlighting the positive correlation between physical stability and sustained biological activity.

Aside from their potential for the delivery of hydrophobic anticancer agents, SLNs also exhibit significant potential for delivering chemically labile compounds that require localized administration. For example, Ghanbarzadeh et al. designed an SLN formulation for the delivery of hydroquinone (HQ), a skin-whitening agent that is highly prone to oxidative degradation [84]. With glyceryl monostearate as the lipid and Tween 80 as the surfactant, HQ-loaded SLNs (HQ-SLNs) exhibited a mean diameter of approximately 86 nm and an encapsulation efficiency of 89.5 %. After five months of storage, the formulation maintained consistent particle size, PDI, and drug content, demonstrating its long-term physical stability (Fig. 3D and E). Of note, although 96 % of HQ was oxidized in aqueous solution, its degradation rate in the SLN formulation was reduced to 31.5 %. In skin permeation experiments conducted using a Franz diffusion cell, as compared to the conventional HQ hydrogel, the HQ-SLN hydrogel significantly reduced transdermal drug transport whilst enhancing dermal accumulation by over three folds (Fig. 3F). These results clearly demonstrate that SLNs can be structurally engineered to protect chemically unstable drugs and improve their localized delivery to skin tissues.

#### 2.2.2 Nanostructured lipid carriers (NLCs)

SLNs attracted attention for their excellent physical stability and low toxicity, as they are composed of solid lipids and biocompatible components [85]. As discussed earlier, SLNs served as effective platforms for addressing the instability of previously developed carriers under physiological conditions [86]. However, they exhibit inherent limitations in terms of structural flexibility due to their rigid solid matrix [87]. Drug molecules are not easily uniformly dispersed within their crystalline lipid core, and over time, lipid rearrangement during storage may cause the drug to migrate to the particle surface or leak out [88]. Such phenomena mostly occur under low-temperature storage conditions and may compromise the consistency of drug release, which is critical for maintaining therapeutic efficacy [89]. Furthermore, the compact crystalline structure of solid lipids limits the internal space available for hydrophobic drug incorporation, reducing the overall drug-loading efficiency [90].

To overcome these limitations, NLCs were proposed as an alternative design [91,92]. NLCs are prepared by blending solid lipids with liquid lipids to partially induce an amorphous internal structure [93]. The resulting irregular arrangement reduces crystallinity and increases the space available for drug loading, enabling stable incorporation of both hydrophobic and hydrophilic drugs [94]. Additionally, the disordered matrix helps achieve gradual drug release without sudden leakage, thereby improving controllability in release profiles [95]. The structurally flexible core also resists external stress, thereby enhancing long-term physical stability [96]. By adjusting the amount of liquid lipid, surface fluidity can be increased, thus changing the exposed lipid composition and surface charge distribution. These changes prevent the formation of fixed binding sites for plasma proteins and weaken hydrophobic and electrostatic interactions [97]. Consequently, non-specific protein adsorption is reduced, and a more stable colloidal state can be maintained under physiological conditions [98]. The emergence of NLCs represents a specific and practical example of structural evolution, wherein enhanced stability and controllability are simultaneously implemented at the design level.

**Fig. 4:**
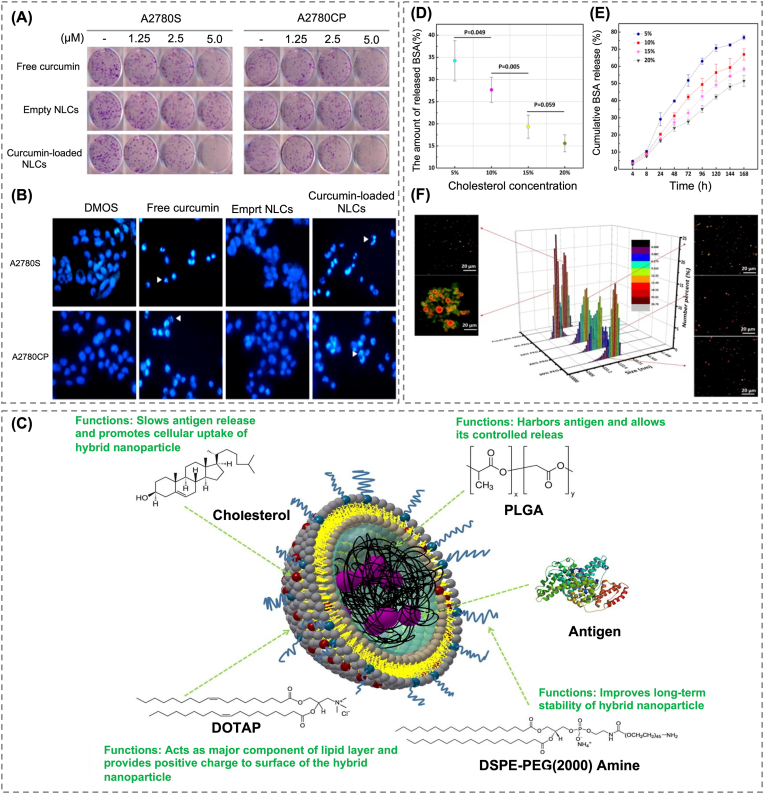
(A) Clonogenic assay demonstrating the suppressive effects of free curcumin, empty NLCs, and curcumin-loaded NLCs on ovarian cancer cell growth. (B) Fluorescence images of Hoechst-stained cells after treatment with DMSO, free curcumin, empty NLCs, or curcumin-loaded NLCs [101]. Copyright 2017, American Chemical Society. (C) Schematic representation of 3D structure of BSA–PLGA nanoparticles coated with biological layer. (D) BSA release from hybrid nanoparticles with varying cholesterol in the lipid layer after 24 h of incubation in 5 % (v/v) human serum at room temperature. (E) The release profile of BSA in PBS (pH 7.4) at room temperature over time. (F) Hybrid nanoparticles containing 20 % cholesterol and DSPE-PEG(2000) amine remained physically stable at 4 °C for 30 days [111]. Copyright 2015, Elsevier.

The partially amorphous internal structure of NLCs allows for more uniform dispersion of hydrophobic drugs rather their confinement to crystalline regions, contributing to both storage stability and controlled release [99]. Bondì et al. encapsulated curcumin, a poorly water-soluble natural anticancer agent, into NLCs and evaluated its delivery performance in ovarian cancer cell lines [100]. The NLCs were prepared using COMPRITOL® 888 ATO as the solid lipid and Captex or Miglyol as the liquid lipid. The resulting particles had an average size of 120–160 nm, a PDI of approximately 0.2, and a zeta potential of −28 mV. These physicochemical properties remained stable after three months of storage. In plasma-based release tests, approximately 30 % of the curcumin was gradually released over 24 h. Of note, NLCs composed of Compritol and Captex exhibited nearly double the cytotoxic effects of free curcumin (Fig. 4A). Through a long-term colony formation assay, free curcumin was shown to suppress A2780S cell colony formation by approximately 50 %, whereas curcumin-loaded NLCs inhibited colony formation by up to 88 %, indicating enhanced anticancer activity (Fig. 4B). Nuclear condensation and fragmentation, as observed through Hoechst staining, further confirmed that curcumin-loaded NLCs preserved the intrinsic apoptotic mechanism of the drug.

The structural advantages of NLCs over SLNs become more evident when they are applied for the delivery of hydrophilic drugs. In a comparative study, primaquine, a water-soluble antimalarial drug, was loaded into both SLNs and NLCs at the same concentration (0.3 % w/v), and NLCs exhibited over twice the drug-loading capacity of SLNs [101]. SLNs composed solely of COMPRITOL ® 888 ATO yielded insufficient space within the crystalline core, whereas NLCs blended with castor oil exhibited inhibited crystal formation and the formation amorphous lipid regions that allowed for more uniform drug distribution. Although both formulations maintained an encapsulation efficiency of approximately 90 %, SLNs exhibited a sharp increase in particle size (exceeding 1 μm) as drug concentration increased, leading to poor dispersion stability. In contrast, NLCs maintained structural consistency, with size variations remaining within approximately 707 nm. These results experimentally support the hypothesis that NLCs offer a high drug-loading capacity and colloidal stability through their internal structural flexibility, even for hydrophilic drugs.

This flexible architecture not only facilitates the encapsulation of individual drugs but also supports the co-loading of agents with distinct physicochemical properties. Chang et al. developed NLCs co-loaded with PTX, a hydrophobic drug, and doxorubicin (DOX), a hydrophilic drug, and evaluated their performance in glioma stem cells (GSCs) [102]. The carriers were composed of COMPRITOL® 888 ATO, oleic acid, and soybean phosphatidylcholine, forming a solid-liquid lipid matrix. Without surface modification, the NLCs exhibited a uniform spherical morphology with an average particle size of 120 nm, a PDI of 0.237, and a zeta potential of −28 mV. Upon hydration, particle size and surface charge remained unchanged. Release studies showed that over 70 % of both drugs were gradually released within 24 h without an initial burst. This sustained release profile suggests that the amorphous matrix ensured uniform drug distribution and prolonged retention. The study demonstrated that the structural characteristics of NLCs alone can support the stable co-encapsulation and controlled release of hydrophobic and hydrophilic drugs without the need for additional surface engineering.

#### 2.2.3 Lipid-polymer hybrid nanoparticles (LPNs)

The structural methods implemented for LBN development have evolved from aiming to control crystallinity and surface flexibility to improving physical stability and functional tunability through the use of more advanced systems. In this context, lipid-polymer hybrid nanoparticles (LPNs), the first formulation based on polymeric materials in lipid-based structures, was developed [103,104]. LPNs merge the compatibility of lipids with the structural versatility of polymers, creating a structural framework requiring a more functionally modifiable carrier [105]. Particularly, LPNs possess a core-shell structure with a polymeric hydrophobic core and a lipid monolayer or bilayer shell [106]. The polymeric core can stabilize the encapsulated drug whilst providing structural stability owing to its rigidity in the described unit. Biodegradable polymers, such as poly(lactic-co-glycolic acid) (PLGA), are commonly used [107]. The phospholipid layer serves as an outer layer that can complex, creating surface properties and providing shell defenses against ambient surroundings [108]. This architecture confers physical stability and greater colloidal dispersity to the carrier [109]. Each component plays a complementary role, providing a basis for structural integrity and controllable release.

To enhance structural stability and minimize drug leakage, researchers have explored lipid–polymer hybrid systems incorporating PLGA as the polymeric core. Hu et al. encapsulated PLGA-entrapped bovine serum albumin (BSA), a hydrophobic protein, within a PLGA matrix and surrounded it with a lipid shell composed of phosphatidylcholine (PC), cholesterol (CH), and DOTAP (Fig. 4C) [110]. The study focused on investigating how variations in cholesterol content affect membrane structure and drug retention. Higher cholesterol concentrations led to increased membrane density, which correlated with reduced drug release. Under serum conditions, the amount of BSA released at 24 h decreased from approximately 34 %–15 % as cholesterol content increased (Fig. 4D). A similar tendency was observed in PBS, with cumulative release over 168 h declining from 77 to 52 %, depending on the lipid composition (Fig. 4E). These results suggest that denser lipid packing can act as a physical barrier that limits unintended drug leakage and improves the structural stability of the carrier. However, excess cholesterol led to particle fusion during long-term storage, resulting in increased particle size and decreased cellular uptake. To prevent this instability, a small amount of PEG-lipid was added to the formulation, and this successfully suppressed particle fusion and preserved particle size during storage (Fig. 4F).

Another LPN design approach focused on stabilizing the particle surface and regulating drug release using a simple phospholipid layer. Thus, lecithin, a naturally derived and biocompatible lipid, was used to form the lipid shell, assembled around a PLGA core [111]. This design aimed to provide basic physical stability whilst maintaining an interface suitable for biological interactions. Nicardipine hydrochloride (NCH), a hydrophilic drug with a short half-life, was selected as the model drug. The resulting formulation achieved an encapsulation efficiency of 92 % and exhibited a zeta potential below −30 mV. The drug release profile of the particles was sustained for 16 days upon evaluation in phosphate buffer saline (pH 7.4). This extended release could not be fully explained by passive diffusion alone. Based on the release profile, the lipid layer appeared to play an active role in modulating drug transport by undergoing subtle structural changes over time. Rather than serving as a passive barrier, the lipid shell contributed directly to the controlled release by adjusting its permeability in response to the surrounding environment. These results indicated that even a simple structural LPN configuration can provide effective release modulation if the lipid composition and interfacial behavior are properly coordinated. This example demonstrates the potential of a minimalistic lipid design in combination with a solid polymeric core for simultaneous provision of physical stability and sustained drug release.

### 2.3 Stage 3: structural adaptations for precision and environmental responsiveness

Although previous advancements in nanocarrier platforms significantly improved release control and structural integrity, these systems still faced significant challenges in biological environments [112]. Various physicochemical conditions reign in target tissues in the body, making it difficult for a fixed structure to perform effectively in all contexts [113]. Nanocarriers frequently fail to reach the intended target, becoming trapped in non-target tissues or remaining in the extracellular matrix without penetrating deep into the lesion [114]. In other cases, even after cellular uptake, carriers could be quickly expelled, limiting their therapeutic efficacy [115]. Premature clearance by the immune system can also significantly reduce the circulation time of the drugs, ultimately compromising treatment effectiveness [116]. These limitations underscore the need for structural strategies beyond simple stability and release control. This has led to the development of more sophisticated design features that allow nanocarriers to escape the immune system, actively bind to cells of interest, and respond to cues in their unique environment, transforming the platform from a passive delivery system to an active one [117,118].

#### 2.3.1 Stealth and long-circulation design

The enhanced permeability and retention (EPR) effect, in which tumor tissues have higher vascular permeability, has made the use of nanoscale drug carriers an attractive approach, particularly in oncology. Delivery carriers, such as nanoparticles, that are approximately 100–200 nm are believed to be optimal because they have a small enough diameter to allow them to leave tumor vasculature via pores (0.1–3 μm) and avoid rapid renal clearance of small diameter of below 6–8 nm [119]. Recently, microfluidic technology has been applied as an effective method to manufacture lipid nanoparticles (LNPs) with consistent size within optimal range. Prolonged circulation is a prerequisite for therapeutic effectiveness; however, commonly used nanocarriers are often rapidly eliminated via hepatic or splenic clearance [120,121]. To enhance immuno-compatibility, surface modification strategies have been employed to avoid immune recognition and reduce non-specific protein adsorption [122]. Among these strategies, PEGylation is a commonly used approach [123]. By attaching polyethylene glycol (PEG) chains to the carrier surface, a hydration layer, that reduces opsonization and recognition by phagocytes, is formed [124]. This leads to extended systemic circulation, improved bioavailability, and enhanced delivery efficiency [125]. PEGylation has been used in various lipid-based platforms, including liposomes, SLNs, and NLCs [126].

Korake et al. formulated PEGylated SLNs for improved systemic retention and tissue distribution of docetaxel (DOC), a hydrophobic anticancer drug [127]. PEG monostearate (SA-PEG2000) was synthesized by reacting stearic acid chloride with methoxy PEG2000, and was incorporated into the SLN surface. This design suppressed off-target protein binding and immune recognition, resulting in improved pharmacokinetics. The PEGylated SLNs reached a maximum plasma concentration of 33.63 μg/mL, higher than that for both non-PEGylated SLNs (25.52 μg/mL) and the free drug (20.56 μg/mL), with a mean residence time of 47.68 h. Distribution analyses also showed decreased accumulation in MPS-associated organs, as well as increased drug concentrations in blood, after administration.

In another study, lipid nanoparticles composed of triglycerol monostearate (TGMS) and DSPE-PEG2000 were developed for *in vivo* delivery of dexamethasone (Dex) [128]. Non-PEGylated carriers exhibited almost complete Dex clearance within 12 h, whereas PEGylated carriers maintained the drug in circulation for over 48 h. Pharmacokinetic analyses revealed that half-life was prolonged four-fold for the PEGylated carriers as compared to the non-PEGylated carriers; in addition, the area under the curve (AUC) was approximately two-fold greater for the PEGylated carriers than for the non-PEGylated carriers. Biodistribution studies using fluorescent lipids also confirmed reduced liver and kidney uptake, and sustained accumulation at inflammation sites. These results indicate that PEG chains effectively prevent early clearance and improve systemic durability.

Following these preclinical findings, LNPs with stealth features have also demonstrated clear potential in clinical applications. A notable example is the BNT162b2 mRNA vaccine developed by Pfizer-BioNTech, in which specific input composition parameters were optimized to obtain a stable and functional LNP design [129]. The formulation consists of ALC-0315 (ionizable lipid), DSPC (phospholipid), cholesterol, and ALC-0159 (PEG–lipid), at a molar ratio of 46.3:9.4:42.7:1.6, respectively. In this formulation, the negatively charged mRNA is electrostatically condensed and protected by the ionizable lipid, cholesterol supports membrane packing and systemic stability, and DSPC provided bilayer stability and fusogenic properties. A PEG–lipid provided a hydrated steric barrier to avoid aggregation and opsonization. Such structural optimization not only yielded long systemic circulation and immunological profile safety benefits, but also allowed for scalable and reproducible manufacturing. This same formulation was assessed in a phase 2/3 randomized, placebo-controlled trial involving 43,448 participants aged 16 years or older [130]. The vaccine group received two doses of 30 μg BNT162b2 intramuscularly 21 days apart (N = 21,720) and the placebo group were given saline placebo (N = 21,728). In the vaccine group, 8 cases of COVID-19 occurred compared to 162 cases in the placebo group, leading to a vaccine efficacy of 95 % (95 % CI, 90.3–97.6). Vaccine efficacy estimates were consistent across age, sex, race, and comorbidity, with 94 % efficacy for participants aged 65 years or older. Safety outcomes were favorable for BNT162b2 with local pain, fatigue, and headache as the most common local and systemic reactions. Serious adverse events occurred in less than 0.5 % of participants, which was consistent with the placebo group. Collectively, these data demonstrated that prolonged circulation and immune safety at the structural level successfully translated into robust and safe protective immunity in humans.

Carrier surface engineering has a critical impact on structural stability and *in vivo* behavior. Thus, surface modification has become a central component of nanocarrier design. In addition to PEGylation, zwitterionic surface coating has emerged as a promising stealth strategy [131,132]. Zwitterions contain both positive and negative charges on the same molecule, enabling the formation of dense hydration shells that resist protein adsorption and immune activation [133]. Zhao et al. introduced zwitterionic lipid nanoparticles (ZwiLNPs) for RNA interference therapy against *PCSK9*, a gene associated with hyperlipidemia [134]. The carrier, the surface of which was coated with DSPE–poly(carboxybetaine) (DSPE–PCB), demonstrated superior hydration and protein resistance properties to PEG-based systems. As compared to their PEGylated counterparts, ZwiLNPs exhibited a prolonged circulation half-life of 6.9 h after the first administration and 10.3 h after repeated dosing. Molecular simulations corroborated this behavior by revealing closer packing of hydration shells, and biodistribution studies demonstrated liver-specific accumulation of siRNA without accelerated blood clearance.

#### 2.3.2 Targeted nanocarriers

Designing nanocarriers with prolonged circulation time has become a foundational strategy for improving drug delivery efficacy as extended systemic retention has been shown to enhance both drug exposure and sustained therapeutic activity across diverse platforms [135]. However, prolonged circulation in the body does not guarantee therapeutic success. Drugs must selectively reach pathological tissues or lesions to exert their intended effects. Off-target distribution not only reduces efficacy but also increases the risk of adverse effects. Therefore, nanocarrier design must aim beyond systemic retention and integrate the entire delivery trajectory, from extracellular transport to intracellular localization, into a structurally coordinated platform [136,137]. Design strategies focus on increasing targeting to the diseased area by attaching ligands, antibodies, or small molecules that can bind specifically to receptors overexpressed on diseased cells [138]. These interactions mitigate side effects by limiting non-specific distribution to off-target sites and ensuring drug activity only at the intended location. Structural design must consider ligand density, orientation, and spatial accessibility, as these factors critically influence targeting efficacy [139,140]. The manner in which nanocarriers are structurally optimized determines not only whether drugs reach their destination but also how effectively they interact with their intended molecular targets.

Depending on their size and surface characteristics, LBNs tend to distribute non-specifically to various hepatic cell types, including hepatocytes, Kupffer cells, and liver sinusoidal endothelial cells [141]. This wide distribution results in reduced delivery efficiency as the drug is diluted in multiple cell types, leading to decreased concentration at the desired target site and increased off-target effects [142]. To circumvent this constraint, Han et al. engineered a lipid nanoparticle system for targeted drug delivery to hepatic stellate cells (HSCs), which are key effector cells in liver fibrosis [143]. They used anisamide, a ligand of sigma-1 receptors overexpressed on activated HSCs and covalently attached it to a specially designed lipidoid (T3A-C12). The siRNA‐loaded LNPs targeted *HSP47*, a fibrosis‐related gene. *In vivo* experiments indicated that anisamide-conjugated LNPs significantly improved siRNA delivery to HSCs, reducing *HSP47* expression by approximately 65 %, and downregulating the expression of fibrotic markers such as α-SMA and collagen I. This is an example of how incorporation of a targeting ligand into a formulation can address non-specific tissue distribution and increase the therapeutic index of RNA-based delivery systems.

**Fig. 5:**
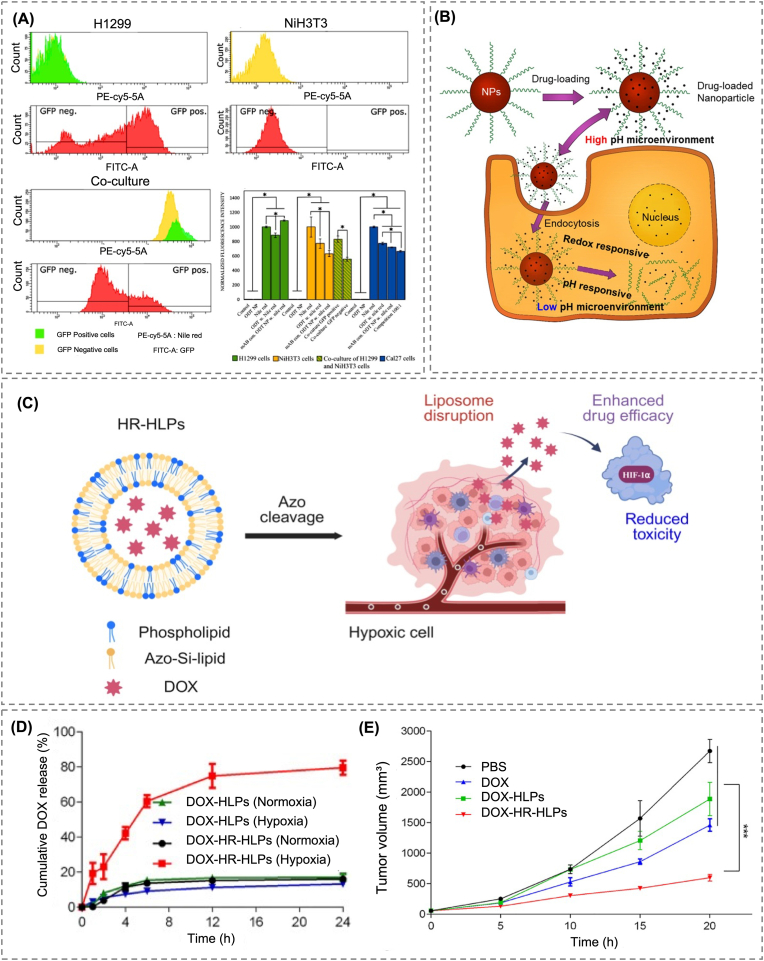
(A) Flow cytometry and co-culture studies demonstrate that anti-EGFR conjugated lipid nanoparticles significantly enhance uptake in EGFR-positive cells while reducing non-specific internalization in EGFR-negative cells, thereby confirming selective targeting capability [144]. Copyright 2021, Elsevier. (B) Schematic illustrating dual-responsive intracellular drug release induced by acidic pH and redox environment, promoting the release of both encapsulated and surface-bound drugs [149]. Copyright 2020, Multidisciplinary Digital Publishing Institute. (C) Schematic illustration of azo-inserted hypoxia-responsive hybrid liposomes, which enable deep penetration into tumors and selective drug release in hypoxic regions co-localizing with nucleus-translocated overexpressed HIF-1α. Created with BioRender.com. (D) Comparison of DOX release between HLP and hypoxia-responsive HR-HLP under normoxic and NADPH-induced hypoxic conditions (mean ± SD, n = 3). (E) Tumor volume progression in BALB/c nude mice showing that DOX-HR-HLPs significantly inhibited tumor growth compared with PBS, free DOX, and DOX-HLPs (mean ± SEM, n = 5, ∗∗∗ p-value <0.001) [138]. Copyright 2020, Elsevier.

In another study, Notabi et al. attached anti-epidermal growth factor receptor (EGFR) antibodies onto the surface of lipid nanoparticles to target EGFR-overexpressing cancer cells [144]. The system was developed using a 1-octadecanethiol (ODT) lipid core and a BSA-based stealth coating, with simultaneous antibody conjugation. Specifically, nanoparticles were produced by solvent injection of ODT (1 mg/mL) into BSA solutions (1–10 mg/mL), yielding particles of ∼137 ± 30 nm with a zeta potential of −29 mV, while DTNB/DTT-mediated disulfide coupling enabled stable antibody attachment. This double-modification strategy reduced non-specific uptake and facilitated selective internalization into EGFR-expressing cells. *In vitro* studies carried out in Cal27 oral cancer cells using Nile red as a model compound demonstrated a 63 % increase in responsive fluorescence for the developed carriers as compared to non-targeted carriers. Similarly, enhanced uptake was observed in H1299 lung cancer cells. In contrast, there was no significant difference in uptake in NIH3T3 cells not expressing EGFR regardless of the presence of antibodies. Competitive binding assays further confirmed EGFR-mediated targeting as free antibody pre-treatment reduced nanoparticle internalization (Fig. 5A). This study illustrated the usefulness of antibody-based active targeting for enhancing the specificity of LNP-based delivery systems for RNA or small-molecule therapeutics.

#### 2.3.3 Stimuli-responsive nanocarriers

Reaching the target site alone does not guarantee therapeutic efficacy if the drug carrier releases its payload without selective responsiveness. A system that releases drugs uniformly, regardless of environmental cues, may cause off-target exposure or fail to activate when needed. For instance, drugs may leak before arrival, exposing healthy tissues unnecessarily, or the delivery system may remain inactive even at the target site, reducing their therapeutic effectiveness [62]. Tumor tissues, which differ from normal tissues in several ways, including acidic pH, reducing conditions, and unique enzymatic activity, present microenvironments that must be considered in the release design [145,146]. Thus, modern drug carriers have evolved to integrate not only targeting capability, but also context-sensitive release, determining when and under what circumstances drug release should occur [147]. This shift has led to the development of systems that respond specifically to tumor-associated signals [148]. Such carriers remain stable under normal conditions but trigger drug release upon the detection of predefined internal cues (Fig. 5B) [149]. In some cases, physical stimuli, such as heat or light, have been employed to enable external control over release behavior [150]. These strategies mark a transition toward carriers that behave dynamically in response to the internal environment of the body, acting as precision platforms rather than passive delivery tools.

Tumor sites are typically hypoxic and rich in reducing agents [151]. Long et al. designed a liposomal system that takes advantage of this by incorporating an azo linkage (–N=N–) into the lipid tail of a phosphatidylcholine derivative (Fig. 5C) [152]. This bond remains intact under normal oxygen conditions but dissociates in reductive settings, causing the liposome to disassemble and release the drug. Using doxorubicin (DOX) as model drug, the liposomes exhibited less than 10 % release under normoxic conditions but over 80 % release under hypoxic conditions (1 % O_2_) within 24 h (Fig. 5D). Fluorescence imaging confirmed that DOX accumulated only in hypoxic regions, which aligned with HIF-1α expression in the nucleus, confirming targeted release. *In vivo* experiments conducted using 4T1 tumor-bearing mice demonstrated that this system increased DOX accumulation in tumors whilst reducing its distribution in healthy tissues, ultimately achieving better tumor suppression than free DOX (Fig. 5E). These findings illustrate how responsive structures can fine-tune both the location and intensity of drug action.

In response to the complexity of tumor microenvironments, dual-responsive systems have been developed. Curcio et al. introduced lipid–polysaccharide nanoparticles (PHYN) capable of releasing drugs when exposed to both acidic pH and high reductive stress conditions, while also targeting CD44 receptors [153]. The system comprised a phosphatidylcholine-based lipid bilayer integrated with oxidized hyaluronic acid (oxHA), and employed disulfide-linked cystamine as a cross-linking framework. Under mildly acidic or reductive conditions, cleavage of imine and disulfide bonds triggered nanoparticle disassembly and drug release. When loaded with DOX, the carrier maintained structural stability under physiological conditions but released over 80 % of its payload under either low pH or high glutathione level conditions. Selective uptake was observed in CD44-overexpressing cancer cells, accompanied by a marked decrease in IC_50_ values as compared to that of the free drug. This design demonstrated how the biochemical features of tumor microenvironments can be structurally embedded to enable targeted and stimulus-responsive delivery.

**Fig. 6:**
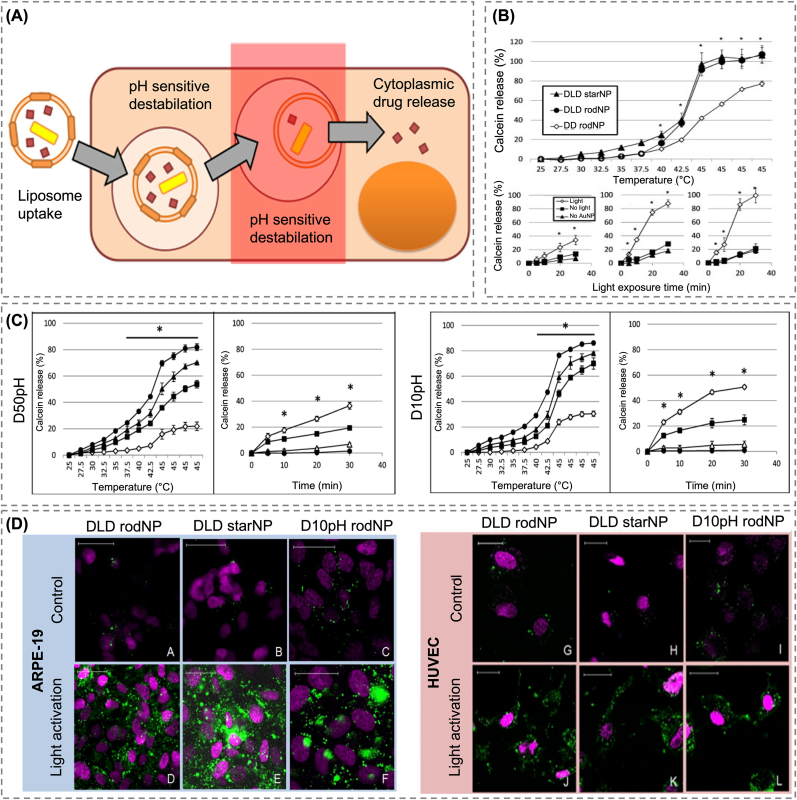
(A) Schematic illustration of light-triggered cytosolic drug delivery via gold nanoparticle-loaded liposomes. Light-induced heat disrupts membranes, enabling controlled release into the cytosol, especially under acidic conditions. (B) Temperature and light-triggered calcein release from gold nanoparticle-loaded liposomes. (C) Calcein was released from D50pH and D10pH liposomes under pH and light-triggered conditions. (D) Intracellular distribution of calcein in ARPE-19 and HUVEC cells treated with DLD or D10pH liposomes, with or without light activation [154]. Copyright 2015, Elsevier. (For interpretation of the references to colour in this figure legend, the reader is referred to the Web version of this article.)

Recent designs have enabled stimuli-responsive nanocarriers to react not only to endogenous tumor cues, but also to externally applied physical stimuli, enabling spatiotemporally controlled drug release. Lajunen et al. developed a liposomal system embedded with gold nanoparticles (AuNPs), which responded to visible and near-infrared (NIR) light via the generation of localized heat, triggering disruption of the lipid bilayer and subsequent drug release into the cytosol (Fig. 6A) [154]. Furthermore, a pH-sensitive lipid composition (diolein/CHEMS) was introduced to facilitate endosomal release under acidic conditions. *In vitro* studies demonstrated minimal release of the payload at neutral pH (7.4), and over 50 % release within 30 min under acidic pH (5.0) conditions upon light irradiation (Fig. 6B). Of the two tested formulations, D10pH, which had a lower diolein proportion, exhibited greater sensitivity to the combined stimuli than D50pH. At pH < 5.0 and temperatures above 42.5 °C, D10pH released over 80 % of its contents, with light-triggered release reaching 51 %, as compared to 37 % for D50pH (Fig. 6C). Confocal microscopic imaging demonstrated that the fluorescent probe (calcein) diffused in the cytosol only after illumination, remaining limited to endosomal vesicles in the absence of light (Fig. 6D). This dual-responsive design is representative of how internal and external stimuli can be combined to achieve highly selective and temporally controlled drug release, further extending the functional flexibility of LBNs for precision therapy.

## 3 Emergence of integrated architectures for multifunctional delivery

Through relatively simple formulations, LBNs with single structural designs have improved drug stability and enhanced pharmacokinetic properties. Surface modifications have also been shown to be effective in enhancing targeting capabilities and biocompatibility. However, structures that serve only one function are limited when confronted with the simultaneous functional demands of biological environments [81,155]. For example, enclosing a drug to protect it from degradation may hinder its timely release, while the addition of targeting ligands can increase binding specificity but also increase serum protein interactions, leading to accelerated clearance [156]. Such functional trade-offs highlight the need for new strategies that can harmonize multiple roles within a single system. Design strategies that spatially separate functional domains or incorporate complementary components within a single carrier have been developed to address the inherent functional limitations of conventional systems. By compartmentalizing activation conditions or combining materials with distinct physicochemical properties, these integrated approaches enable the concurrent operation of multiple functions without interference. This leads to an increase in structural complexity, allowing for more refined control over therapeutic behavior. This section presents representative examples that illustrate how such designs have resolved functional trade-offs and improved delivery outcomes [105]. Structural sophistication has been achieved either by isolating operational conditions to prevent functional interference or integrating components with distinct characteristics to compensate for individual limitations [157]. This section further examines how such integrated architectures have addressed inter-functional constraints and enhanced delivery performance through representative case studies.

### 3.1 Multi-compartment systems

When drug carriers are required to handle compounds with differing release profiles or stability conditions, a single-compartment design often proves inadequate [158]. For example, one drug might need to be released quickly, while another might need to be continuously exposed. Similarly, to maintain the stability and bioactivity of each compound, co-loading hydrophilic and hydrophobic compounds necessitates spatial separation [159]. If compartmentalization and activation control are not correctly implemented, premature release of one agent can interfere with the performance of the other agents, especially in stimuli-responsive environments [160]. Structural compartmentalization has become a successful design approach for overcoming these obstacles, allowing for the independent control of several functions and improving delivery accuracy in challenging therapeutic conditions [161].

**Fig. 7:**
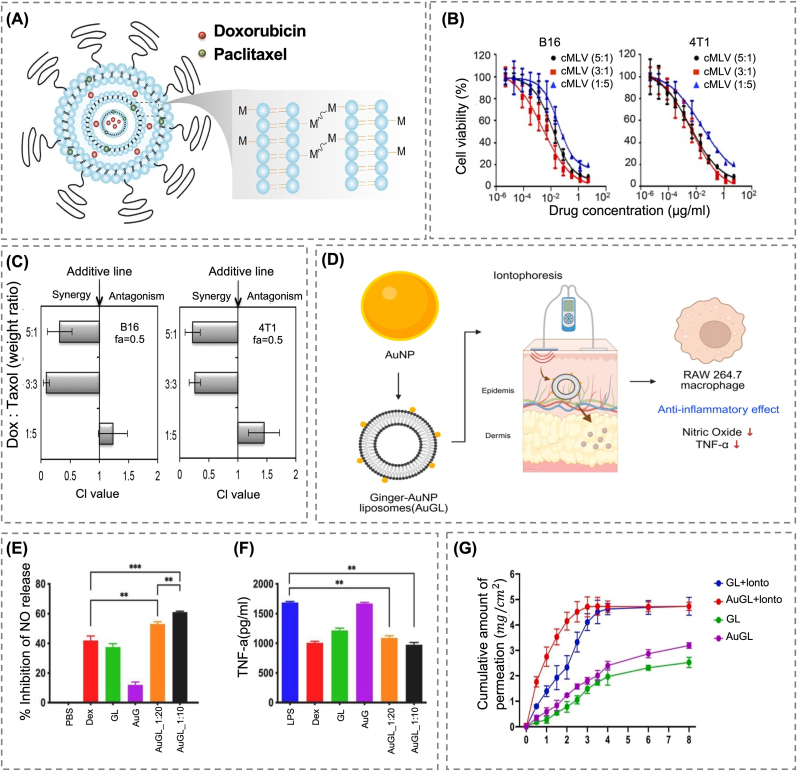
(A) Schematic representation of the cMLV structure enabling co-delivery of hydrophobic and hydrophilic drugs. (B) Cytotoxicity analysis of cMLV-mediated co-delivery of Dox and PTX at different weight ratios (5:1, 3:3, 1:5) in B16 and 4T1 cells. (C) Combination index (CI) of cMLVs evaluated at a survival fraction of 0.5 [162]. Copyright 2014, American Chemical Society. (D) Schematic illustration of AuGLs, where gold nanoparticles are tethered onto ginger-loaded liposomes to enhance iontophoresis-mediated transdermal penetration and to exert anti-inflammatory effects by reducing nitric oxide and TNF-α production in macrophages. Created with BioRender.com. (E) Quantitative analysis revealed differences in NO production, reflecting the anti-inflammatory effects of each treatment group. (F) Comparative assessment of TNF-α levels following treatment with various samples; dexamethasone was used as the reference inhibitor. (∗∗ p < 0.01, ∗∗∗ p < 0.001). (G) The cumulative release profiles of gingerol from various liposomal formulations, with and without iontophoresis, are illustrated in the graph [169]. Copyright 2024, Elsevier. (For interpretation of the references to colour in this figure legend, the reader is referred to the Web version of this article.)

Liu et al. engineered a cross-linked multilamellar vesicle (cMLV) capable of co-encapsulating DOX and PTX, chemotherapeutic agents with distinct pharmacological profiles and ratio-dependent synergy (Fig. 7A) [162]. The multilayered structure spatially separated PTX and DOX within the lipid bilayers and aqueous layers, respectively. The structure was enhanced through Mg^2^+-mediated fusion and chemically crosslinked using dithiothreitol, allowing for independent loading and release. *In vitro*, cMLVs with Dox:PTX ratios of 5:1 and 3:3 significantly enhanced cytotoxicity as compared to free drugs or their combination at mismatched ratios, with a marked decrease in IC_50_ and enhanced synergistic activity observed in B16 melanoma and 4T1 breast cancer cells (Fig. 7B and C). In contrast, the 1:5 ratio formulation induced phosphorylation of extracellular signal-regulated kinase (ERK), indicating the activation of pro-survival signaling pathways. *In vivo* experiments demonstrated the primary therapeutic impact of the optimized cMLV, including effective tumor suppression, improved survival, a stable intra-tumoral drug ratio for at least 24 h under various treatment conditions, and significantly reduced cardiotoxicity. Overall, this study demonstrated how spatially compartmentalized designs can achieve fine-tuned control over drug synergy and dosing ratio, providing compelling evidence for the therapeutic application of multi-compartment delivery systems.

Spatial compartmentalization can also be appropriate for single-drug systems when control of timing of action or triggering conditions is necessary. This could be achieved by using an external physical trigger to activate discrete compartments in a more complex vesicular structure. Kong et al. developed a pressure-sensitive multivesicular liposome (PSMVL) designed to release its payload in response to elevated pulmonary arterial pressure, a pathological condition associated with high-altitude pulmonary edema (HAPE) [163]. The hydrophilic vasodilator, amlodipine besylate (AB), was compartmentalized into multiple aqueous domains, enabling rapid release once a defined pressure threshold was exceeded. The lipid matrix was composed of DPPG (4.2 mg/mL), DEPC (37.2 mg/mL), and cholesterol (21.4 mg/mL), with tricaprylin incorporated at 1.2 mg/mL to confer pressure sensitivity, yielding vesicles of ∼27 ± 2 μm with a zeta potential of −34.7 ± 1.8 mV and an encapsulation efficiency of ∼78 %. The carrier adopted a DepoFoam-like architecture in which pressure sensitivity varied by compartment location, with tricaprylin asymmetrically embedded to weaken interlamellar cohesion and induce selective rupture. Release studies demonstrated that while both carriers sustained release under standard conditions, AB-PSMVLs exhibited an initial burst release of 32.7 % within 0.5 h at pressures above 25 mmHg, with cumulative release up to 50.5 % at higher pressure stimulus. PSMVLs were fabricated via a W/O/W double emulsion approach, followed by reverse-phase evaporation and freeze–thaw cycling, resulting in a structure responsive to pathological conditions. This configuration minimized non-specific leakage and allowed for drug release only when and where needed. *In vivo* studies in a mouse model of HAPE demonstrated significant AB accumulation in lung tissues, improved blood oxygen saturation (SpO_2_), suppressed pulmonary edema, and ultimately, downregulated HIF-1α expression. Furthermore, the system facilitated repeatable release based on serial stimulation, proving that PSMVLs can be adaptable to dynamic disease environments and exemplify the functional utility of stimulus-responsive compartmentalized carriers.

### 3.2 Hybridization of distinct material properties for enhanced delivery

Carriers composed of a single material often lack the versatility required to concurrently fulfill critical demands such as structural robustness, biological activity, and targeted delivery [164]. To overcome functional limitations that cannot be solved with single-material carriers, recent strategies have consisted of incorporating structural elements with different physicochemical properties [165]. For example, some systems integrate the mechanical strength of polymers with the biocompatibility of lipids or combine the controllability of inorganic materials with the stimuli-responsiveness of organic components [[166], [167], [168]] The integration of structurally and functionally distinct materials is a strategic approach for preserving the unique functions of each component, minimizing mutual interference, and enabling precise coordination of the diverse requirements of drug delivery.

Sansanaphongpricha et al. designed a ginger gold-loaded liposome (AuGL) system, wherein gold nanoparticles were tethered to the liposomal surface to overcome the limitations of iontophoretic skin permeation [169]. Although liposomes exhibit excellent biocompatibility and drug-loading capacity, their low surface electron density limits their applicability in electrically stimulated delivery. To address this limitation, the researchers physically tethered highly conductive gold nanoparticles onto the outer surface of liposomes with the aim of improving their responsiveness to electrical stimuli and enhancing both transdermal penetration and cellular uptake (Fig. 7D). The AuGL was constructed using *Zingiber officinale* extract, a hydrophobic herbal ingredient, as model drug. A gold:liposome ratio of 1:10 was identified as the optimal condition for ensuring both structural integrity and biological safety. *In vitro* tests on RAW 264.7 showed that the carrier exerted anti-inflammatory effects by suppressing TNF-α and nitric oxide production (Fig. 7E and F). When loaded with Nile red for fluorescence imaging, the AuGL exhibited an over two-fold enhancement in cellular uptake as compared to conventional liposomes. Additionally, the formulation was transformed into an emulgel, merging the rheological properties of both emulsions and gels, and assessed for skin penetration using a porcine skin model. Under electrical stimulation conditions, drug permeation significantly increased with respect to that of the non-stimulated controls, indicating that the formulation itself contributed to the enhanced delivery performance (Fig. 7G). These findings indicate that the combination of structurally distinct components, such as gold nanoparticles and liposomes, yields drug delivery outcomes that would be challenging to achieve using a single-component carrier.

Prompted by the persistent challenges associated with low drug bioavailability and limited retention time on the ocular surface, Yu et al. designed a composite delivery system (NLC-Gel) that integrated nanostructured lipid carriers (NLCs) with a dual-responsive hydrogel [170]. Although NLCs provide high permeability for hydrophobic drugs, as well as lipid-based biocompatibility, they are prone to rapid clearance following topical instillation, limiting their sustained action. Conversely, hydrogels offer strong muco-adhesion and prolonged retention, but are limited by poor corneal permeability. To reconcile these contrasting characteristics, the researchers embedded baicalin (BN)-loaded NLCs into a hydrogel matrix, composed of carboxymethyl chitosan and Poloxamer 407, which responds to both pH and temperature changes. Genipin, a natural crosslinker, was used to improve structural cohesion and ensure biocompatibility. The resulting BN-NLC gel exhibited an initial burst release followed by sustained drug diffusion, achieving an approximately 4.5-fold higher corneal permeability than conventional eye drops. Rheological and swelling studies validated the response of the system to the external environment, with prolonged residence time on the ocular surface. Fluorescence imaging also demonstrated increased cellular uptake and resistance to washing, and irritation testing showed minimal toxicity and stable hydration, underscoring its suitability for ocular use. Through the functional integration of carriers with distinct physicochemical characteristics, this system successfully achieved drug persistence, permeability, and safety levels that are rarely attainable with single-phase structures.

## 4 Expansion of structural design for strategic functional implementation

The LBN structure has long served as the primary interface between it and the biological environment, playing a central role throughout the delivery process, from stabilization and targeting, to controlled release [1]. More recently, variables originating outside the carrier have become increasingly influential in guiding structural design. Thus, drug carriers are no longer regarded as fixed physical constructs but as flexible components that can be tailored within integrated therapeutic frameworks [171]. This transition is exemplified by the emergence of next-generation delivery platforms, wherein structural strategies are systematically aligned with therapeutic objectives, such as organ-specific targeting, immune modulation, and controlled activation. Table 4 summarizes these approaches, highlighting how architectural features, ranging from organ-selective lipid compositions to AI-guided lipid engineering, are leveraged to meet complex biological demands. Recent studies reflect this perspective well. For example, Selective Organ Targeting lipid nanoparticles (SORT-LNPs) have shown that lipid composition can be adjusted to achieve lung or spleen selectivity beyond the liver by modulating protein corona formation and endocytic pathways [172,173] Logic-gated liposomes respond to intracellular signals such as ATP abundance and membrane protein recognition to trigger fusion, thereby implementing release only under combined conditions within targeted cells [174] In addition, engineered multi-domain LNPs have been proposed to modularize structural, interfacial, payload, and environmental elements, thereby coordinating responses to heterogeneous tumor microenvironments and complex delivery routes. Programmable LNPs extend modular design into a predictive framework in which lipid composition can be adjusted according to modeled distribution profiles, enabling spatiotemporal control of tissue selectivity and release [175,176].

**Table 4:** Structural strategies and therapeutic features of next-generation drug delivery systems.

Type	Delivery mechanism	Target sites	Therapeutic effect	Limitations	Reference
SORT LNP	Organ-selective distribution via combinatorial design of ionizable and SORT lipids	Liver, Lung, Spleen	Tissue-specific delivery and reduction of off-target accumulation	Off-target accumulation in non-intended organs; limited cross-species reproducibility	[172]
DNA Logic-Gated Liposome	Conditional membrane fusion triggered by ATP or membrane protein sensing	ATP-rich cells, Tissues overexpressing target proteins	Targeted liposome-cell fusion and selective anticancer agent release	Complex synthesis; limited stability in physiological conditions	[174,234]
Engineered Multi-Domain LNP	Integration of multiple functional domains to enable complex delivery routes	Tumor microenvironments, Inflamed tissues, Endothelial barriers	Enhanced target selectivity, stimuli-responsive release and structural stability;	High structural complexity; increased cost; reproducibility issues	[235]
Exosome-Hybrid LNP	Biomimetic uptake through exosome-mediated cellular recognition and internalization	Tumors, Immune-related tissues	Improved targeting and biocompatibility; reduced systemic toxicity	Source heterogeneity; non-standardized CMC for large-scale production	[[236], [237], [238]]
Hybrid MSC-EV LNP	Fusion with mesenchymal stem cell-derived extracellular vesicles for immune evasion and targeting	Inflammatory tissues, Tissue interfaces, Liver	Enhanced biocompatibility; prolonged circulation; improved targeting	Immune variability: scalability of EV sourcing remains challenging	[239,240]
Programmable LNP	Lipid composition designed based on predictive models of organ-specific distribution	Liver, Spleen, Muscle	Tunable delivery efficiency and precision targeting of specific tissues	Reliance on predictive modeling; limited algorithm accuracy	[241,242]
AI-Optimized Ionizable-Lipid LNP	AI-guided quantitative modulation of delivery efficiency via optimized ionizable lipid design	Liver, Lung, Lymph nodes	Increased delivery efficiency; minimized toxicity; efficacy at low doses	Dataset dependence; need for prospective validation in clinical settings	[243,244]

Design strategies are increasingly focused on determining the sequence, method, and mechanism through which carriers react to changing *in vivo* conditions, suggesting that structural design now functions not merely as a means of implementing discrete functions but as a platform for organizing the entire delivery strategy. This section explores the systematic arrangement of structures of drug carriers around key strategic principles in modern therapeutic design. Particular attention is given to the coordination of structural responses across multi-stage delivery routes, the biomimetic reconstruction of biological systems, and AI-enhanced predictive approaches for engineering responsive architectures.

### 4.1 Structural design reflecting multistep biological barriers

Following administration, drug delivery carriers must follow a sequential route through the body, encountering various biological barriers. They must preserve their structural integrity, avoid fast clearance, arrive at target tissues, traverse cellular membranes, and ultimately reach their intracellular targets [13,177]. Each step involves distinct environmental constraints, necessitating design strategies that reflect these changing conditions [178]. To successfully navigate this intricate biological environment, carriers must be structurally optimized not only for specificity to the target, but also for performance adaptability throughout the delivery process.

A recent example of such a design is the high-efficiency protein LNP developed by Ren et al. [179]. Ionizable LNPs were structurally tailored to enhance endosomal escape, which is a key requirement for effective cytosolic delivery of protein therapeutics. Generally, cationic LNPs form stable complexes with anionic proteins via electrostatic interactions but often lack the features for efficient endosomal escape. To enable charge conversion under acidic conditions and promote endosomal disruption, the researchers combined a fixed-charge cationic lipid, DOTMA (1,2-di-O-octadecenyl-3-trimethylammonium propane), with a pH-responsive ionizable lipid, DLin-MC3-DMA (heptatriaconta-6,9,28,31-tetraen-19-yl 4-(dimethylamino)butanoate), at a 1:1 M ratio. This lipid combination was further blended with DOPE, cholesterol, and DMG-PEG2000, resulting in a five-component system specifically engineered to traverse various biological barriers. DOPE facilitated membrane fusion for endosomal escape, cholesterol enhanced structural stability during circulation, and DMG-PEG2000 provided steric stabilization to reduce non-specific interactions in the bloodstream. Following systemic administration, the LNPs spontaneously bound to serum albumin, allowing for tissue-selective uptake through gp30/gp18-mediated caveolae-dependent endocytosis, effectively circumventing traditional clearance pathways and improving cellular entry. This strategy was further validated using a galectin-8 recruitment assay, which provided real-time evidence of MC3-mediated endosomal disruption. Functional delivery of saporin and IL-10 subsequently confirmed therapeutic efficacy, with both tumor cell apoptosis and enhanced immune activation observed. By implementing compartmentalization based on biological challenges across the delivery route and integrating distinct functions within a single carrier architecture, this system exemplified a structurally coordinated response to the multistep barriers of protein transport, culminating in positive therapeutic outcomes.

Zhang et al. developed trastuzumab-conjugated chitosan-iodoacetamide-coated SN-38-loaded liposomal nanoparticles (TZ-SN-CsIA LNPs) as a precision delivery system for HER2-positive lung cancer treatment [180]. This system was designed using a stepwise structural strategy that directly responded to the biological barriers encountered throughout the delivery process. The chitosan derivative (CsIA) reversed the negative surface charge of liposomes to a positive charge, improving stability in circulation and enhancing membrane affinity. Its coating also reduced premature drug leakage whilst allowing for sustained release under acidic conditions, thereby maximizing drug effectiveness within the tumor microenvironment. Additionally, conjugation with trastuzumab, a monoclonal antibody specific for HER2, enabled selective targeting through receptor recognition. The formulation exhibited high uptake and significant intracellular accumulation in Calu-3 lung cancer cells, and its potent anticancer effects were confirmed via AO/EB and DAPI staining. This study introduced an LNP platform structurally optimized to address charge conversion, pH-responsive release, and antibody-mediated specificity in a coordinated manner, integrating precision, persistence, and selectivity into a single system.

Wang et al. developed blood-brain barrier (BBB)-penetrating lipid nanoparticles (BLNPs) for the systemic delivery of mRNA to the central nervous system [181]. The BLNPs were prepared from MK-series of ionizable lipids, DOPE, cholesterol, and DMG-PEG2000. Initial screening was performed with the BLNP made from MK6/DOPE/Chol/DMG-PEG2000 at a 20/30/40/0.75 (molar ratio) and the optimal formulation was MK16/DOPE/Chol/DMG-PEG2000 at a 60/30/40/0.75 and a lipid-to-mRNA mass ratio of 12.5:1. The resulting BLNPs had a hydrodynamic diameters of ∼140 nm, PDI <0.15, and encapsulation efficiencies of ∼83 % and a mildly positive zeta potential. Mechanistic studies indicated that caveolin inhibition, cholesterol extraction, and γ-secretase inhibition reduced BBB passage indicating caveolae-mediated transcytosis was the primary mechanism. BLNPs given intravenously at 0.5 mg kg^−1^ mRNA had widespread distribution throughout the brain with high protein expression in astrocytes and neurons while minimal uptake in MPS organs. For the MK series, MK6E at the 12.5:1 ratio improved about 2-fold higher brain bioluminescence compared to the MK6 formulation, with alkyl-tail modification leading to MK16. The MK16 provided ∼1.7-fold higher expression than MK6E, and ∼8.3-fold higher than MC3-based LNPs at the same doses and without disproportionally higher distribution to the periphery. PTEN mRNA delivery given via MK16 BLNPs in glioblastoma models had the potential for high anti-tumor activity, with approximately 70 % of treated mice surviving beyond 120 days. In summary, these studies illustrate systematic lipid optimization that enabled the BLNPs to escape the systemic circulation, cross the BBB, and achieve parenchymal gene expression, a structural approach to multi-step biological barriers.

### 4.2 Biomimetic nanocarriers inspired by physiological architectures

As DDSs have evolved into actively functioning therapeutic platforms within the body, structural design has progressively incorporated strategies to enhance biocompatibility and dynamic interactions in complex physiological environments [182]. In this context, biomimetic approaches that emulate or reconfigure the structural and functional components of biological systems have garnered increasing attention in DDS design [183]. These strategies are derived from intricately organized biological architectures that perform specific physiological functions; these systems are now advancing beyond mere superficial likeness to mimicking how living systems react to stimuli and regulate functions at the structural level [184]. For example, nanocarriers coated with natural cell membranes, which mimic cellular functions, such as signal detection, molecular transport, and immune system modulation, can avoid immune clearance and enhance tissue-specific accumulation through self-recognition [185]. Similarly, nanocarriers that utilize or mimic biological structures involved in intercellular communication, such as exosomes, tend to exhibit superior adaptability and targeting specificity *in vivo* as compared to conventional synthetic nanoparticles [186]. Nonetheless, extracellular vesicle (EV)-based carriers, such as exosomes face intrinsic source-dependent heterogeneity and scale-up challenges. In contrast, synthetic peptide mimics are more reproducible yet lack the complex protein and glycan repertoire of natural vesicles [187,188]. As the integration of biological functions that were previously difficult to reproduce in artificial carriers becomes increasingly feasible, DDSs are gradually evolving towards dissolving their boundary with the biological environment and functioning as part of its physiological processes [189].

A representative example of this approach is cell membrane coating (CMC), a surface modification strategy that enhances nanoparticle biocompatibility whilst overcoming the limitations associated with conventional ligand-based modifications, such as immune activation [190]. Membranes sourced from various cell types, including red blood cells, cancer cells, and macrophages, can be fused onto the nanoparticle surface while preserving their native protein, lipid, and glycan composition, thereby conferring multiple biological functionalities, such as immune evasion, prolonged circulation, and homotypic targeting [191]. However, the reproducibility of CMC systems is strongly influenced by donor cell variability, making standardized sourcing and scalable preparation critical requirements for successful clinical translation.

For instance, Zhou et al. developed NLCs coated with red blood cell membranes (RBCm-PTX-NLCs) to circumvent the side effects and rapid clearance associated with PTX [192]. This delivery system retained the characteristic protein profiles of RBCm, including CD47, which allowed for macrophage-mediated phagocytosis evasion; *in vivo* tumor distribution analyses further confirmed its capacity for immune evasion and extended circulation. Coumarin-6-based cellular uptake and drug release assays demonstrated efficient tumor cell internalization and significant drug retention. In addition, analyses conducted using the S180 tumor model demonstrated enhanced tumor accumulation, improved therapeutic efficacy, and prolonged survival. These findings indicated that RBCm coating can simultaneously confer various delivery functions essential for effective *in vivo* performance, including immune evasion, targeting accuracy, and systemic stability.

**Fig. 8:**
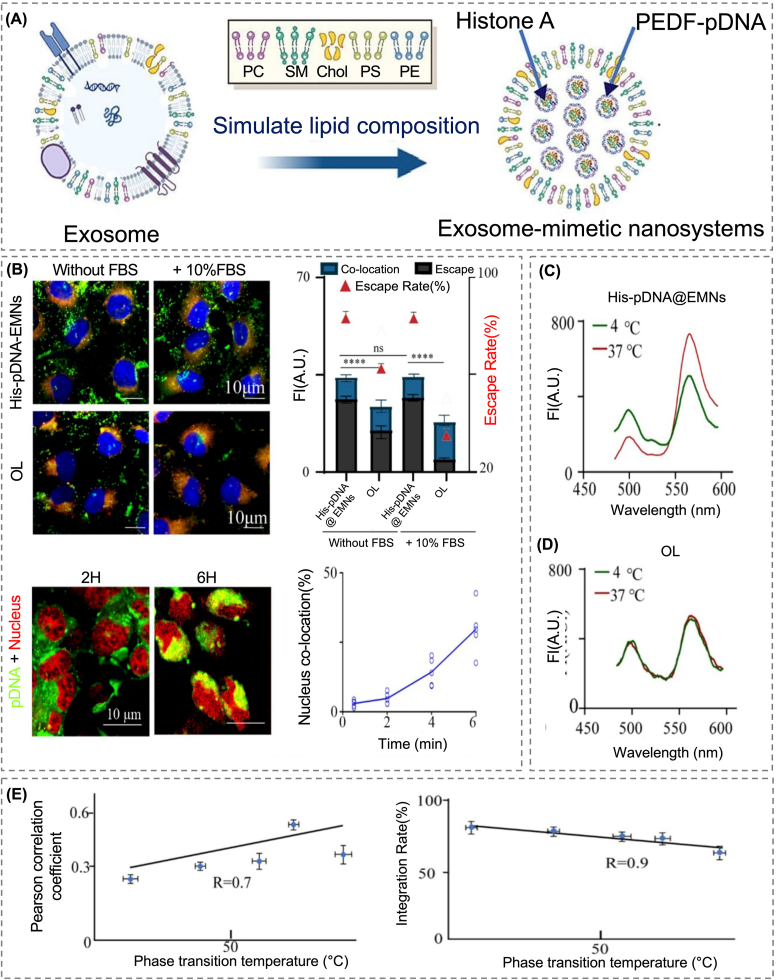
(A) Schematic illustration of exosome-mimetic nanovesicles loaded with histone A and PEDF-pDNA. (B) Confocal microscopy images demonstrate efficient lysosomal escape and time-dependent nuclear localization of His-pDNA@EMNs in HUVECs, confirming intracellular delivery of PEDF-pDNA. (C) FRET spectra of His-pDNA@EMNs at 4 °C and 37 °C after 1 h of lysosomal interaction, and (D) corresponding spectra of OL under the same conditions, showing minimal energy transfer change, suggesting improved membrane fusion by His-pDNA@EMNs. (E) Correlation analysis between the phase transition temperature of EMNs and the efficiency of their membrane fusion with lysosomes [193]. Copyright 2025, Elsevier.

As an extension of cell-derived structural mimicry, studies have reported the development of artificial exosome-mimetic delivery systems designed to closely replicate the lipid composition and surface protein organization of natural exosomes. Li et al. designed exosome-mimetic nanovesicles (EMNs) by recreating a lipid and functional protein composition that closely resembled that of natural exosomes for the prevention and treatment of high-altitude pulmonary edema [193]. The formulation consisted of the key exosomal lipids, sphingomyelin (SM), phosphatidylcholine (PC), phosphatidylserine (PS), phosphatidylethanolamine (PE), and cholesterol, at optimized ratios. Histones were also incorporated to condense and protect DNA, allowing for the stable encapsulation of the gene encoding the pigment epithelium-derived factor (PEDF) (Fig. 8A). His-pDNA@EMNs exhibited typical exosomal features, including a particle size of 102 nm, a surface charge of −20 mV, and a distinctive cup-like morphology. After cell entry through clathrin-mediated endocytosis, the vesicles successfully escaped lysosomal degradation and reached the nucleus within a few hours, resulting in efficient gene expression (Fig. 8B). Each lipid played a distinct role in the delivery process: PC facilitated cellular entry, PE and PS aided in endosomal escape, and SM and PS supported nuclear translocation. Upon inhalation, His-pDNA@EMNs selectively accumulated in pulmonary endothelial cells and triggered PEDF expression (Fig. 8C and D). In an HAPE mouse model, the EMNs improved oxygen saturation and decreased pulmonary edema and vascular permeability, indicating their therapeutic efficacy (Fig. 8E). This study demonstrated that lipid- and protein-based biomimicry can offer a reliable strategy for enhancing both the efficiency and safety of gene delivery.

Another biomimetic platform is the lipoprotein mimic, which aims at replicating the three-dimensional architecture of lipid-protein complexes found in nature. Based on this approach, He et al. developed a biomimetic system by creating nanoparticles that emulated the composition and structural characteristics of high-density lipoproteins (HDL), and named it, biomimetic HDL (bHDL) [194]. The particles were created using phospholipids, the primary lipid component of HDL, and D4F, a synthetic peptide that mimics apolipoprotein A-I function. The fabricated nanoparticles exhibited a uniform diameter of approximately 15 nm with a slightly positive zeta potential of 5–8 mV, closely matching the surface profile of native HDL. Protein composition was strictly defined using the D4F peptide at a controlled peptide-to-lipid ratio of 1:50 (w/w), minimizing batch-to-batch heterogeneity compared to cell-derived exosomes. Overall, the final structure closely resembled native HDL in both morphology and nanoscale organization. The system was designed to target kidney injury molecule 1, a highly expressed receptor in damaged renal tubular epithelial cells, for selective accumulation at sites of renal injury. Aside from exhibiting the properties of a passive drug carrier, bHDL also exhibited the intrinsic physiological traits of HDL, such as lesion-specific delivery, long circulation time, and immune evasion. The platform achieved a synergistic therapeutic effect in suppressing renal fibrosis through the co-delivery of triptolide and nintedanib. Developed based on the engineering of the functions and structure of endogenous lipoproteins, bHDL overcame the limitations of conventional delivery approaches, including poor accumulation efficiency and systemic toxicity.

### 4.3 Precision-driven structural design via predictive modeling

The structural design of DDSs has begun shifting toward more intricate and flexible configurations with the deepening of understanding on biological barriers. Specifically, the architecture of next-generation DDSs is continuously expanding toward finely-tuned coordination with the physiological environment [195,196]. Recently, increasing efforts have been directed towards integrating different types of biological information into the early stages of design. This includes data on target physiology, tissue function, and metabolic conditions [[197], [198], [199]]. In this process, artificial intelligence and deep learning-based predictive models offer the possibility of analyzing complex relationships between biological variables and directly applying the results in carrier structure design [200]. Prediction-based precision design is garnering attention as a key technology for refining the logical basis for structural design and enabling the implementation of patient-specific delivery strategies [201].

**Fig. 9:**
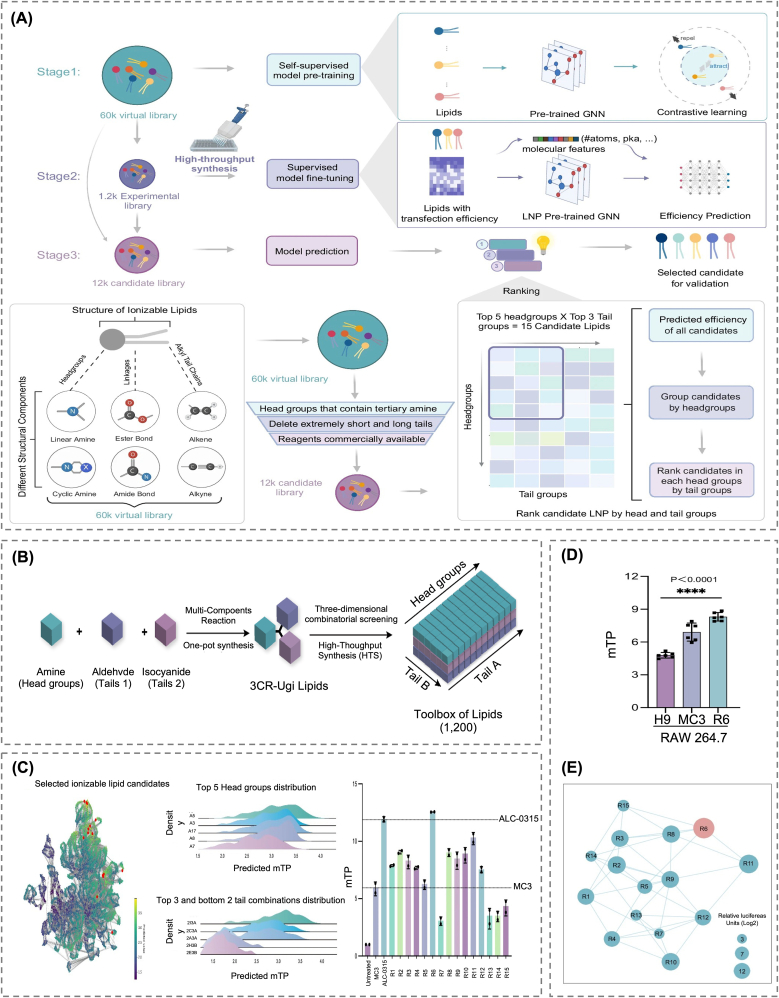
(A) Schematic overview of the AGILE platform showing the workflow from virtual lipid library construction and model training to high-throughput synthesis, candidate filtering, and selection of top ionizable lipids. (B) Schematic illustration of the high-throughput synthesis approach used to generate a library of 1200 ionizable lipids. (C) UMAP projection, structure-based clustering, and experimental validation identified highly effective ionizable lipids for mRNA delivery in RAW 264.7 cells. (D) Comparative evaluation of mRNA transfection potency (mTP) of H9, MC3 and R6 based LNPs in RAW 264.7 macrophages (n = 6). (E) Network visualization of the top 15 ionizable lipid candidates identified in RAW 264.7 cells, categorized by structural similarity [202]. Copyright 2024, Springer Nature.

Xu et al. proposed a strategic platform that integrates combinatorial chemistry with deep learning-based prediction models to optimize the design of lipid nanoparticles for mRNA delivery [202]. This approach, called, AGILE (AI Guided Ionizable Lipid Engineering), began with the establishment of a virtual library of approximately 60,000 ionizable lipids. A self-supervised learning process was implemented using graph neural networks (GNN) for the exploration of this large chemical space, followed experimental synthesis and validation (Fig. 9A). Then, the model was fine-tuned using experimental data from 1200 synthesized lipids, each tested for its mRNA transfection potency (mTP) (Fig. 9B). The trained model enabled rapid screening of candidate lipids by predicting their delivery efficiencies and helped identify optimal structures tailored to different cell types. One of the lead candidates, H9, showed selective mRNA delivery to muscle tissue, with lower liver toxicity and improved tissue specificity as compared to ALC 0315, a standard lipid component used in current mRNA vaccines. To identify efficient lipids for delivery to RAW 264.7 cells, the model predicted mTP values for approximately 12,000 lipid candidates. These results were visualized using dimensionality reduction techniques, such as UMAP, for the distinction of high-performing structures, and structure-based clustering further supported the identification of effective ionizable lipids in RAW 264.7 cells (Fig. 9C). Among the top 15 predicted lipids, R6 outperformed both MC3 and H9 in terms of transfection efficiency within RAW 264.7 cells (p < 0.005, n = 6) (Fig. 9D). The potency values obtained in HeLa cells correlated strongly with *in vivo* intramuscular delivery, with a Pearson correlation coefficient of 0.78 and a correlation of 0.756 in C2C12 cells. In addition, H9 reduced hepatic accumulation and serum ALT/AST levels while enhancing muscle expression, whereas R6 enabled macrophage selective delivery with limited off-target effects. Assessment of the structures also ultimately indicated R6 had significant structural similarities with a number of the other best candidates via structure-motif repetition, and delivery metrics (Fig. 9E). These findings indicate that AGILE offers a predictive framework rooted in rational structure-function relationships.

Efforts to incorporate AI-based predictive modeling into the structural design of delivery systems are no longer limited to mRNA platforms, but are also expanding to systems for small-molecule therapeutics. A recent study reported the development of self-assembled nanoparticles loaded with the anticancer drug, sorafenib, using fucoidan and polyethyleneimine as base materials. The optimization process integrated artificial neural networks (ANNs) with a design of experiments (DoE) framework [203]. The researchers employed a central composite design to quantitatively evaluate how structural variables, such as polymer ratio, influence particle size and drug encapsulation efficiency. Multiple machine-learning models, including ANN, support vector machine, lasso, k-nearest neighbors, and bootstrap forest, were compared. Among these, an ANN–DoE approach achieved the highest accuracy and reproducibility in predicting optimal formulation conditions. Nanoparticles fabricated under these optimized conditions exhibited a mean diameter of approximately 280 nm, as well as a stable structure. The system enabled the sustained and controlled release of sorafenib in an acidic environment (pH 5.0). Hemocompatibility assays showed a hemolysis rate below 5 %, indicating good biocompatibility. Furthermore, as compared to free sorafenib, the optimized nanoparticles exhibited a significantly enhanced anticancer effect in MDA-MB-231 breast cancer cells, with an IC_50_ of 2.017 μM. This study highlights the potential of integrating AI-driven modeling with experimental optimization for quantitative guidance of the structural design of nanocarriers and for simultaneous enhancement of drug release characteristics and therapeutic performance. Such integration offers a foundational strategy for the rational and predictable development of precision drug delivery systems.

## 5 Conclusion

The structure of DDSs has progressed in response to the intricate challenges encountered in biological settings. While early carriers served merely as protective barriers to stabilize drugs and mitigate physicochemical limitations, modern systems have evolved into sophisticated platforms capable of controlled release, targeted delivery, and environment-specific responsiveness. This transformation is not simply a by-product of technological progress, but reflects a strategic design process aimed at meeting the multilayered functional requirements encountered *in vivo.* Contemporary DDSs are no longer defined by the sum of their technological components; instead, they operate as strategic units that determine not only what functions a system performs, but also when, where, and how these functions are activated. From establishing the sequence of functional engagement to determining how each component contributes to overall therapeutic outcomes, the structure of nanocarriers is essential for the coordination of these components.

In this review, structure has been considered not as a passive vessel of function, but as a tangible expression of design intent, shaped to address specific biological barriers. With a focus on LBNs, known for their biocompatibility and architectural versatility, we have discussed how structural strategies have been applied to overcome physiological barriers and improve delivery efficiency. Overall, the fundamental design principle derived from this analysis is that a systematic understanding of the biological barriers, determined by the route of administration and target tissue, and the development of tailored structural strategies to address them, are essential for designing effective DDSs. This process of structural adaptation can be suggested as an evolutionary response of DDS to the challenges posed by biological barriers. Furthermore, the important point is that these multistep requirements can sometimes conflict with one another, which makes it necessary to recognize such trade-offs and implement structural compromises that enhance overall delivery efficacy. Rather than focusing on each barrier in isolation, this perspective highlights an integrated strategy that views the entire delivery pathway as a unified process. For example, the EPR effect favors nanoparticles in the 50–200 nm range, yet smaller particles penetrate tumors more deeply. At the same time, excessively small carriers face the risk of rapid clearance, which means that effective tumor-targeted design requires balancing these competing demands.

Despite remarkable progression, key limitations still remain. In particular, achieving complete target specificity is extremely difficult. Regardless of how refined the structural design may be, non-specific distribution and unintended exposure are inevitable to some extent, which can give rise to cumulative toxicity during long-term or repeated treatments [204]. Furthermore, even within the same disease, the characteristics of biological barriers can vary among patients and disease stages, making it difficult to guarantee optimal performance across all clinical scenarios through standardized structural design alone. In particular, changes in vascular permeability and interstitial fluid pressure, which differ with disease progression and patient-specific tumor microenvironments, exert a significant influence on the intratumoral distribution and penetration of nanoparticles [205,206]. Tumor heterogeneity exacerbates these concerns. Even within a single tumor, variations in vascular architecture, interstitial pressure gradients, and cellular density make uneven microenvironments that result in heterogeneous delivery efficiency of identical carriers across different regions [207,208]. Another major challenge is the adaptability of the immune system. Repeated administrations can induce accelerated blood clearance, during which nanocarriers become increasingly recognized and eliminated, resulting in a gradual decline in treatment efficacy [209,210].

To solve these problems, the design of next-generation DDSs requires strategies beyond the incorporation of new materials or technologies. The priority is to reinforce structural adaptability so that carriers can dynamically adjust to changing biological environments and patient-specific conditions. This approach emphasizes the importance of personalized design necessitating the development of diagnostic technologies for rapid assessment of patient-specific barriers and modeling platforms capable of predicting optimal structures. Ultimately, in DDSs, structure is not merely a physical framework but the central interface that mediates and regulates complex interactions with the biological environment. The key to successful delivery lies in identifying the barrier characteristics that vary with administration routes, target tissues, and disease states, and systematically operationalizing them into structural strategies. In this way, structural design must be regarded as a strategic tool for interpreting and harnessing biological complexity, and as a foundation for advancing precision therapeutics.

## CRediT authorship contribution statement

**Yeeun Woo:** Writing – original draft, Visualization, Validation. **Jinwook Yoon:** Writing – original draft, Visualization, Data curation. **Yoseph Seo:** Writing – review & editing, Conceptualization. **Yunseon Han:** Writing – review & editing. **Hah Young Yoo:** Writing – review & editing. **Hiesang Sohn:** Writing – review & editing. **Min-Ho Lee:** Writing – review & editing. **Taek Lee:** Supervision, Project administration, Funding acquisition, Conceptualization.

## Declaration of competing interest

The authors declare that they have no known competing financial interests or personal relationships that could have appeared to influence the work reported in this paper.

## Data Availability

No data was used for the research described in the article.
